# Cancer Genomics Identifies Regulatory Gene Networks Associated with the Transition from Dysplasia to Advanced Lung Adenocarcinomas Induced by c-Raf-1

**DOI:** 10.1371/journal.pone.0007315

**Published:** 2009-10-08

**Authors:** Astrid Rohrbeck, Jürgen Borlak

**Affiliations:** 1 Department of Molecular Medicine and Medical Biotechnology, Fraunhofer Institute of Toxicology and Experimental Medicine, Hannover, Germany; 2 Center for Pharmacology and Toxicology, Hannover Medical School, Hannover, Germany; Lehigh University, United States of America

## Abstract

**Background:**

Lung cancer is a leading cause of cancer morbidity. To improve an understanding of molecular causes of disease a transgenic mouse model was investigated where targeted expression of the serine threonine kinase c-Raf to respiratory epithelium induced initialy dysplasia and subsequently adenocarcinomas. This enables dissection of genetic events associated with precancerous and cancerous lesions.

**Methodology/Principal Findings:**

By laser microdissection cancer cell populations were harvested and subjected to whole genome expression analyses. Overall 473 and 541 genes were significantly regulated, when cancer versus transgenic and non-transgenic cells were compared, giving rise to three distinct and one common regulatory gene network. At advanced stages of tumor growth predominately repression of gene expression was observed, but genes previously shown to be up-regulated in dysplasia were also up-regulated in solid tumors. Regulation of developmental programs as well as epithelial mesenchymal and mesenchymal endothelial transition was a hall mark of adenocarcinomas. Additionaly, genes coding for cell adhesion, i.e. the integrins and the tight and gap junction proteins were repressed, whereas ligands for receptor tyrosine kinase such as epi- and amphiregulin were up-regulated. Notably, Vegfr- 2 and its ligand Vegfd, as well as Notch and Wnt signalling cascades were regulated as were glycosylases that influence cellular recognition. Other regulated signalling molecules included guanine exchange factors that play a role in an activation of the MAP kinases while several tumor suppressors i.e. Mcc, Hey1, Fat3, Armcx1 and Reck were significantly repressed. Finally, probable molecular switches forcing dysplastic cells into malignantly transformed cells could be identified.

**Conclusions/Significance:**

This study provides insight into molecular pertubations allowing dysplasia to progress further to adenocarcinoma induced by exaggerted c-Raf kinase activity.

## Introduction

The leading cause for lung cancer is cigarette smoking. More than 80% of lung cancer patients either actively smoke or have been smoking in the past even though other factors such as exposure to asbestos, radon, and genetic factors may contribute to disease as well. Notably, at the time of diagnosis about one third of the patients are already in an advanced stage IV of disease therefore limiting curative therapeutic options, while 15–20% of patients exhibit signs of disease at an early stage. Tumors are classified by morphological appearance and are divided into small cell (SCLC) and non-small cell lung cancer (NSCLC), with NSCLC accounting for approximately 75–80% of all lung cancers. This group is divided further into squamous cell (35–40%) and adenocarcinomas (30–40%), where as the large cell and the undifferentiated non-small cell lung cancers account for the remaining subtypes [1].

Despite intensive research the molecular events essential for the development of lung adenocarcinomas remain elusive, even though it is well established, that the disease involves activation of oncogenes. For instance, K-ras mutations are detected in 20–30% of adenocarcinomas and inactivation of tumor suppressor genes, such as p53, p16INK4a, and Rb are frequently observed as well [2], [3], [4], [5]. Defects in DNA repair pathways and cell cycle checkpoints allow tumor cells to accumulate mutations that are advantageous to growth, invasion, and spread. Of considerable importance is the abnormal expression of growth factors, their receptors, and their signalling pathways that may result in unrestrained cell division. For instance, the transmembrane receptor EGFR (epidermal growth factor receptor) is expressed at high levels in adenocarcinomas [6]. EGFR and its ligands, the neuregulins initiate a signal transduction cascade involving the mitogen-activated protein kinase pathway that constitutes a growth stimulatory loop in lung cancer [7]. Additionaly, high telomerase activity was reported for primary NSCLC particularly at advanced stages of disease while in normal quiescent cells telomerase activity is not detectable. There is a need to better understand the molecular basis of tumorigensis and microarray studies are instrumental in the decoding of the lung cancer genom. Recently, we reported the molecular characterisation of lung dysplasia in a c-Raf transgenic mouse model that develops lung adenocarcinomas [8], [9]. Specifically, c-Raf is a serine/threonine proteine kinase and a direct downstream effector of Ras. It is activated to its GTP-bound state in response to various ligands through binding to their cognate receptors, and is involved in several signaling cascades. Undue activation of Raf signalling is a key event in lung adenocarcinoma and this mouse model recapitulates the genetic events associated with the different stages of tumor development, i.e. from low to high grade dysplasia to highly and less differentiated adenocarcinomas. There is also evidence for Raf-1 overexpression in human lung adenocarcinomas [10] and in a recent report c-Raf was shown to antagonize apoptosis induced by IFNα in human lung cancer cells [11], [12].

In our recent study we focused particularly on the regulatory gene networks associated with dysplasia and identified some molecular reactions that were consider as priming factors for malignant transformation. We now report findings of approximately 12 month old transgenic mice to gain further information at advanced stages of disease, i.e. beyond dysplasia. By use of laser microdissection pressure catapulting (LMPC) tumor cells without any contamination with normal epithelia, blood vessels, stromal cells and tumor necrosis could be isolated. Genome wide expression analyses of tumor lesions were than compared with transgenic but otherwise normal cells or non-transgenic cells. We combined microdissection with gene expression profiling to determine differentially expressed genes as to identify the gene regulatory networks associated with adenocarcinoma. We identified groups of genes acting in concert that were specifically associated with malignant transformation and we identified candidate genes and pathways that likely contribute to progression from lung dysplasia to cancer. Overall, this study identified several regulatory gene networks specifically associated with the progression from dysplasia to malignantly transformed respiratory epithelium.

## Results

### Histological changes

**Figure 1:**
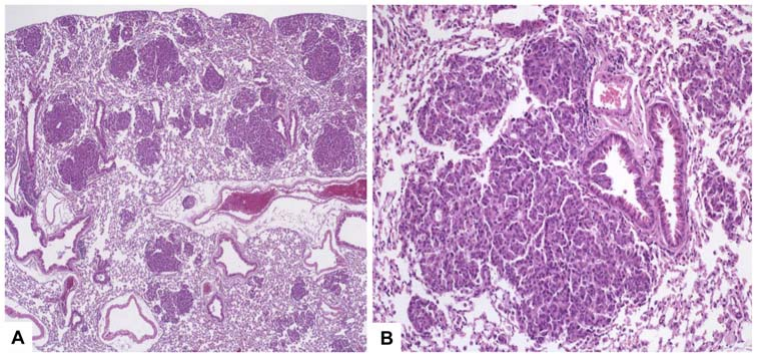
Histological analyses of lung tissue from 12-month-old transgenic mice. Histological analyses of lung tissue from mice transgenic for lung-targeted expression of the cRaf-1 protein. Lung tissue from a 12-month-old SP-C-c-Raf mouse were sectioned at 10 µm, fixed in methanol/acetig acid and stained with H&E. We detected multiple foci of adenocarcinoma. A: Overview presentation (magnification: ×50), B: adenocarcinoma (magnification: ×200).

As shwon in Figure 1, twelve month old animals display multifocal tumor growth and typical features of lung adenocarcinomas. The morphological abnormalities relate to cell structure, number of cells, and cytological appearance of the epithelium. Specifically, the typical bronchioloar columnar epithelium with vertically oriented nucleus is replaced by tumor cells. The tumors cells are columnar to polygonal with high nuclear to cytoplasmic ratios, marked pleomorphism, and prominent nucleoli. Abundant mitotic activity but also tumor associated apoptosis is observed.

### SAM (Significance Analysis of Microarrays)

**Figure 2:**
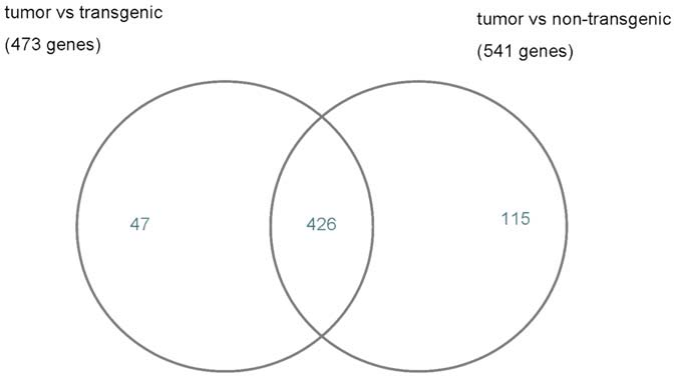
Venn diagram for significantly regulated genes. Venn diagram for significantly differential expressed genes. Comparison of tumor and transgenic unaltered lung tissue with non-transgenic samples. 426 genes were found in tumor, respectively, which were at least 2-fold differentially expressed (FDR = 0.001).

When transgenic but otherwise unaltered lung cells were compared with cancer cells a total of 473 significantly regulated genes (48 up-regulated and 425 down-regulated) were determined (Supplementary Table S1). In a further comparison of non-transgenic lung cells with cancer cells 113 up-regulated and 428 down-regulated genes could be identified (Supplementary Table S2). For such comparison, we requested at least 2-fold differentially expressed genes at an estimated false discovery rate  = 0.001 (Table 1). Transgenicity alone was associated with 16 up-regulated and 2 down-regulated genes (Supplementary Table S3). We further searched for differentially expressed genes by comparing the data sets of adenocarcinoma vs transgenic and adenocarcinoma vs non-transgenic. In such a comparison 426 genes were regulated in common (Figure 2).

**Table 1:** Differentially expressed genes (FDR = 0.001, FC ≤2≥).

	non-transgenic	transgenic	tumor
**non-transgenic**	0	18	541
**transgenic**	18	0	473
**tumor**	541	473	0

### Principal component analysis (PCA) and hierarchical gene cluster analysis (HCA)

**Figure 3:**
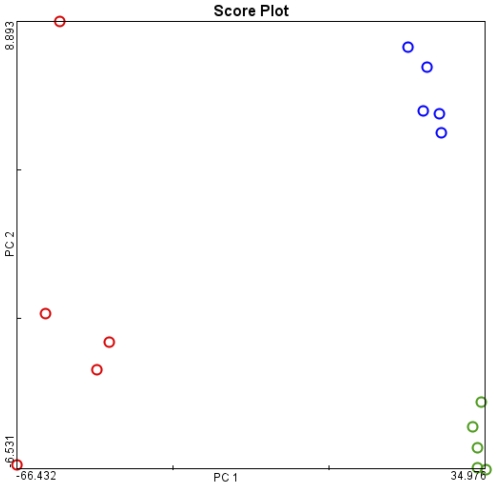
Principal component analysis for gene expression profiles of transformed cells. Principal component analysis of cancer cells from transgenic SP-C/c-raf mouse model in comparison to unaltered lung tissue of transgenic and non-transgenic mice. red, tumor; blue, transgenic non-tumor sample; green, non-transgenic samples.

The expression levels were analyzed by GCOS (GeneChip Operating Software) and the ArrayTrack software. We initial examined the data in a 34.000 genes ×15 samples matrix. The PCA segregated the data into 3 major groups, namely non-transgenic, transgenic and adenocarcinoma (Figure 3).

**Figure 4:**
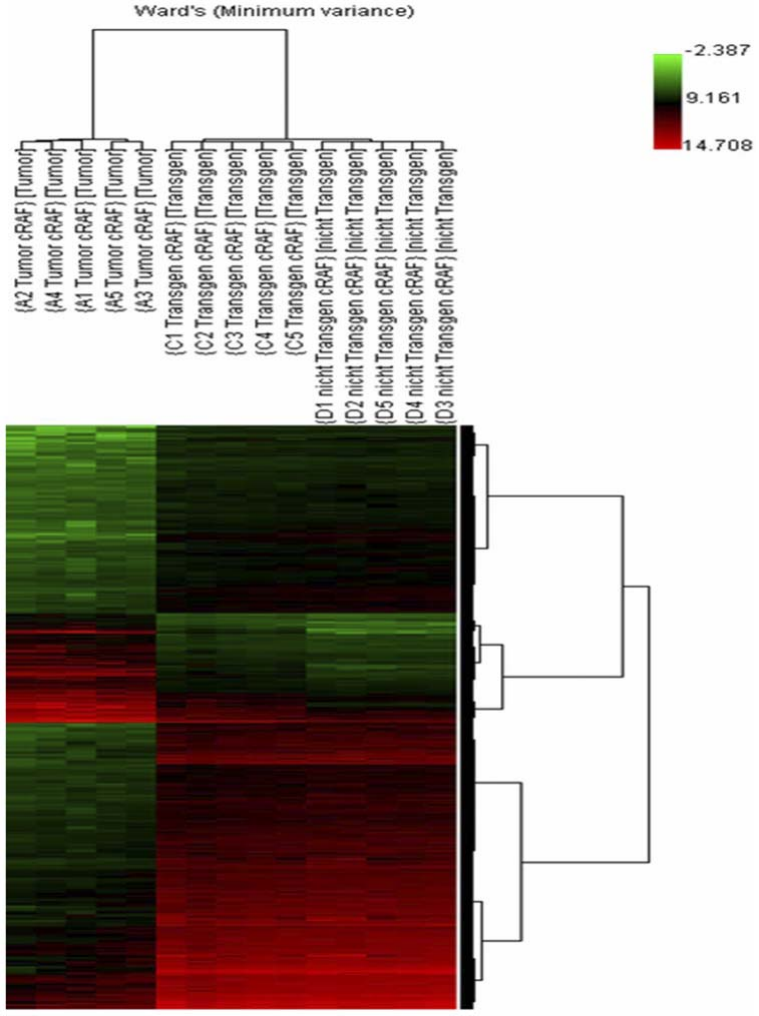
Result of the hierarchical cluster analysis. The normalized data were used for the Ward's Minimum Variance linkage clustering algorithm. A total of 3246 differentially expressed genes (mean channel intensity >100, FDR: 0.1, Bad Flags: 5) were used in the cluster dendogram to obtain a clear segregation of the analyzed groups (tumor, transgenic and non-transgenic). Expression values were colour coded with a red green scale. Green, transcript levels below the median; black, equal to the median and red, greater than median.

We also performed hierarchical gene cluster analyses and searched for the closest pair of expression values of 3246 differentially expressed genes that grouped together. Consequently the data are organized in a phylogenetic tree in which the branch lengths represent the degree of similarity between the values. A clear segregation of the analyzed data (adenocarcinoma, transgenic but unchanged lung tissue and non-transgenic lung tissue) was obtained (Figure 4) and the PCA and HCA grouped data according to their biological state. Correspondingly, gene expression data of transgenic SP-C/c-Raf lung were well separated from the non-transgenic and adenocarcinoma cells, suggesting a large difference between these groups. Stringent cut-offs were used. With an estimated false discovery rate of 0.1 we obtained in adenocarcinoma 2727 significantly regulated genes (895 up-regulated and 1832 down-regulated) compared with transgenic but unaltered cells and 3765 genes (1401 up-regulated and 2364 down-regulated) in adenocarcinoma versus non-transgenic cells. Comparison of these data sets resulted in 2631 genes regulated in common in adenocarcinoma (data not shown).

### Pathway analysis of significantly expressed genes

**Figure 5:**
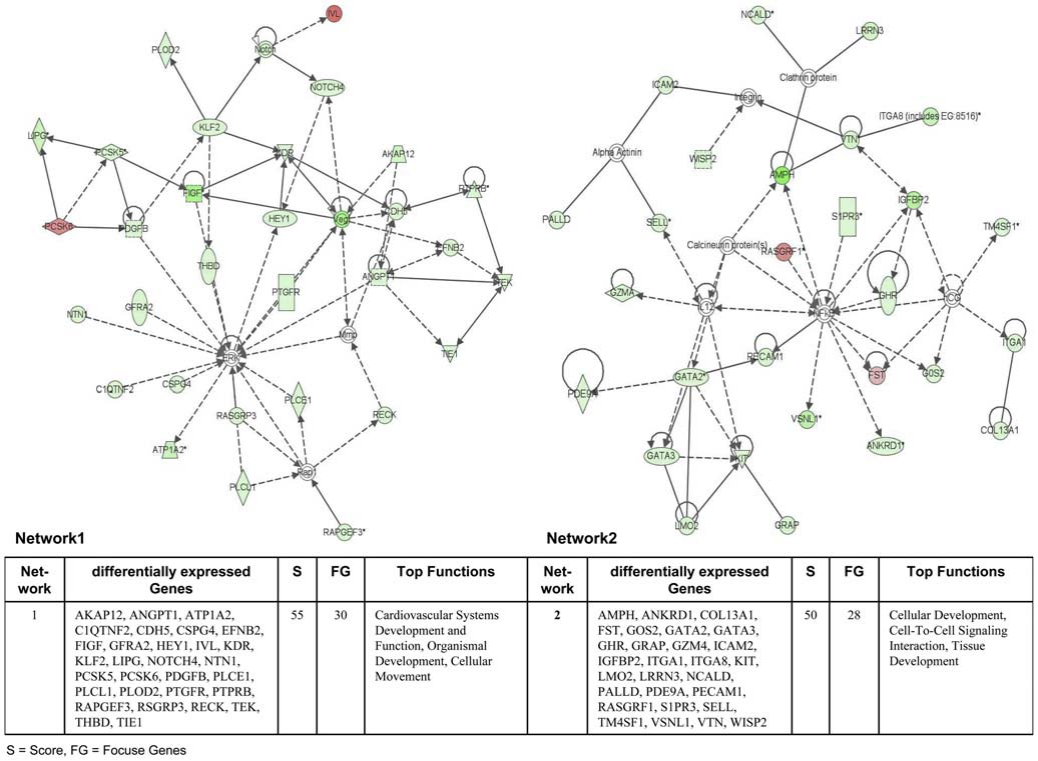
Ingenuity networks: adenocarcinoma versus transgenic mice. Ingenuity networks generated by mapping the focus genes that were differentially expressed between tumor and transgenic unaltered lung tissue (part I). Each network is graphically displayed with genes/gene products as nodes (different shapes represent the functional classes of the gene products) and the biological relationships between the nodes as edges (lines). The length of an edge reflects the evidence in the literature supporting that node-to-node relationship. The intensity of the node color indicates the degree of up- (red) or downregulation (green) of the respective gene. A solid line without arrow indicates protein-protein interaction. Arrows indicate the direction of action (either with or without binding) of one gene to another. IPA networks were generated as follows: Upon uploading of genes and corresponding fold-change expression values (done separately for tumor vs transgenic and tumor vs non-transgenic differentially expressed genes), each gene identifier was mapped to its corresponding gene object in the IPA Knowledge Base (part of the IPA algorithm). Fold-change expression values were used to signed genes whose expression was differentially regulated; these “focus genes” were overlaid onto a global molecular network contained in the IPA Knowledge Base. Networks of these focus genes were then algorithmically generated based on their connectivity and scored according to the number of focus genes within the network as well as according to the strength of their associations.

**Figure 6:**
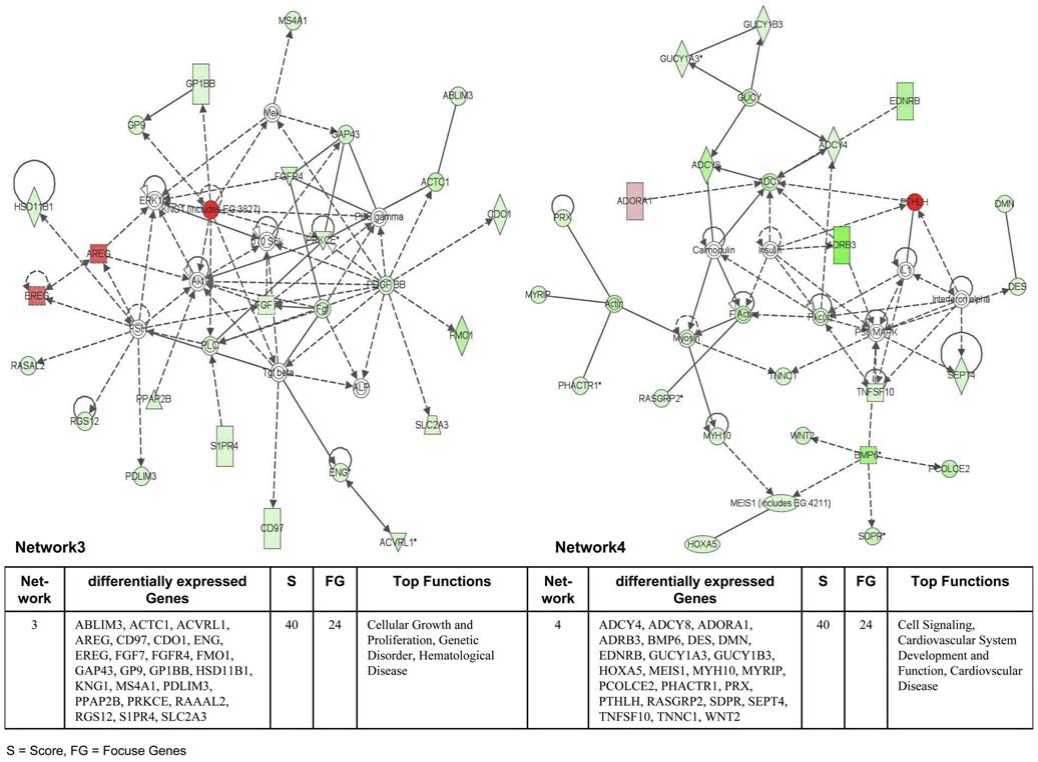
Ingenuity networks: adenocarcinoma versus transgenic mice. Ingenuity networks generated by mapping the focus genes that were differentially expressed between tumor and transgenic unaltered lung tissue (part II).

**Figure 7:**
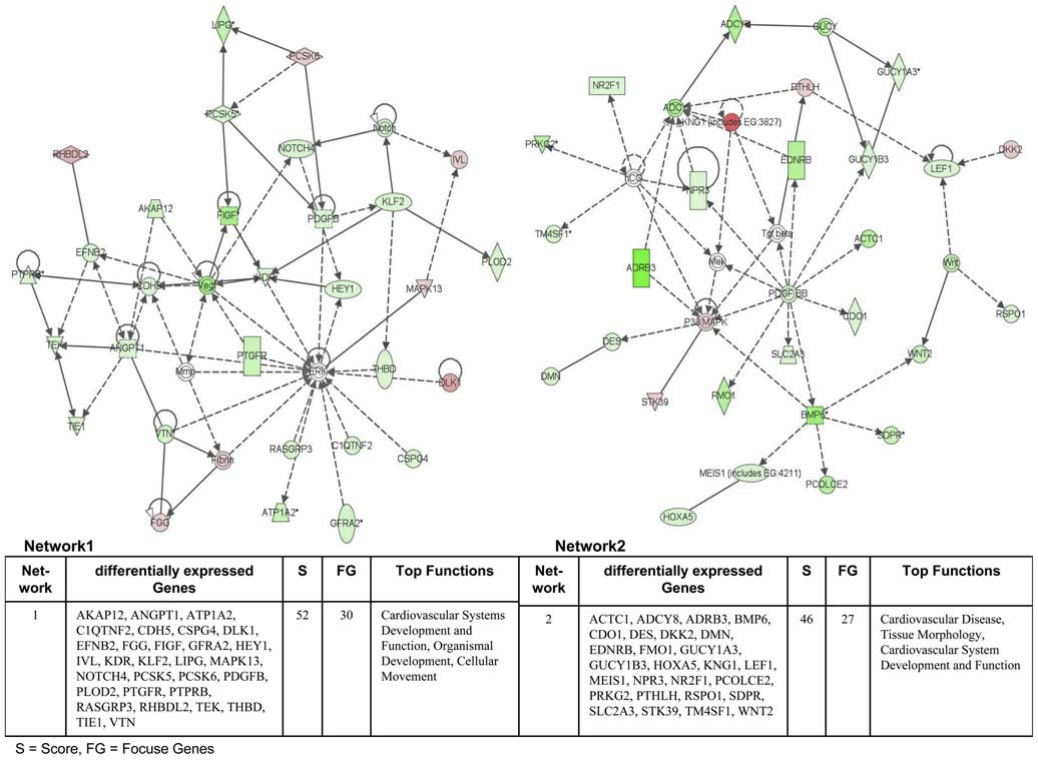
Ingenuity networks: adenocarcinoma versus non-transgenic mice. Ingenuity networks generated by mapping the focus genes that were differentially expressed between tumor and non-transgenic unaltered lung tissue (part I).

**Figure 8:**
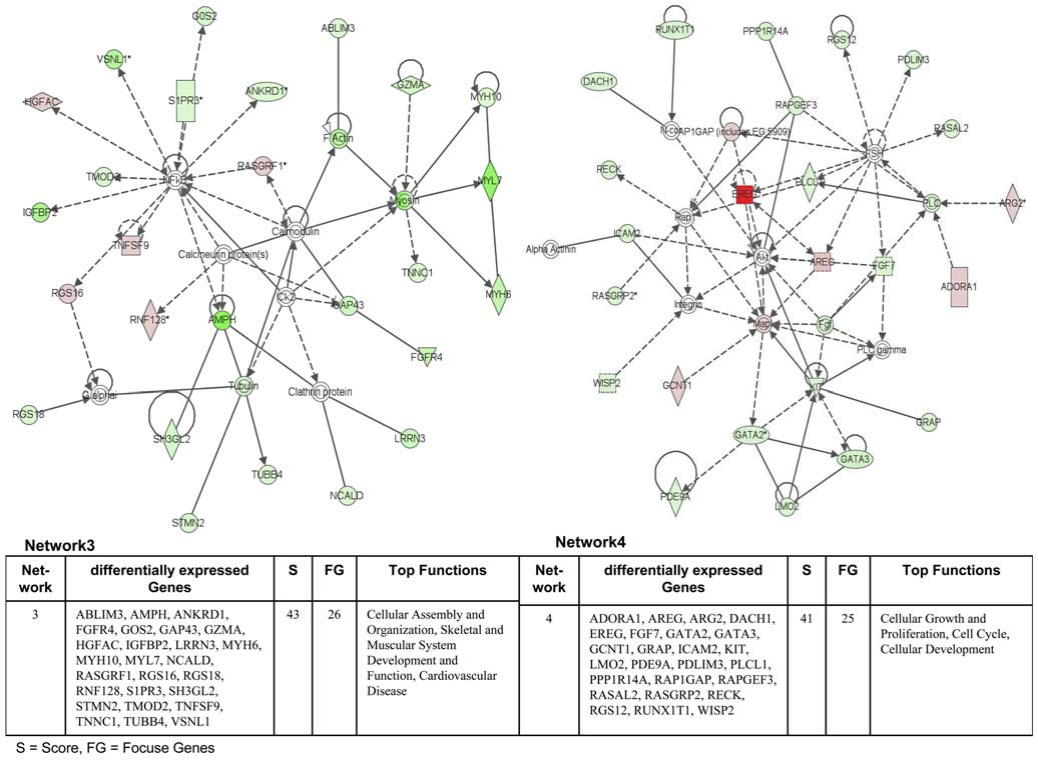
Ingenuity networks: adenocarcinoma versus non-transgenic mice. Ingenuity networks generated by mapping the focus genes that were differentially expressed between tumor and non-transgenic unaltered lung tissue (descriptions see Fig. 6) (part II).

The Ingenuity Pathways Analysis (IPA) software was employed and over 75% of regulated genes were mapped to different networks in the IPA database. These networks describe functional relationships among gene products based on findings presented in peer-reviewed scientifically validated biological pathways. Taken collectively, 24 and 30 networks could be defined for the comparison adenocarcinoma vs transgenic and adenocarcinoma vs non-transgenic cells. Based on pathway analysis the top 4 networks reached a score of 40 or higher and contained 20 or more genes in each of the networks in the comparison adenocarcinoma vs transgenic cells (Figure 5 and 6) and in adenocarcinoma vs non-transgenic cells (Figure 7 and 8). This demonstrates the extensive relationship and interaction between the significantly regulated genes in lung cancer. The networks were specifically associated with cell signalling, cell adhesion, proliferation and growth, development and lipid metabolism (Supplementary Figure S1 and S2). In the following, we wish to describe prominent examples.

### Aberrant cell signalling

We searched for genes involved in cell signalling and found 6 genes to be up-regulated in adenocarcinoma ranging between 5.9 to 28.1-fold as well as 36 genes to be down-regulated ranging between −3.8 to −29.4-fold when transgenic but morphologically unaltered lung tissue was compared. Likewise 5 genes were up-regulated between 7.4 to 30.0-fold and 42 genes were down-regulated (−4.1 to −35.2-fold) when compared with non-transgenic lung tissue. In particular, Pthlh (parathyroid hormone-like hormone protein) was 28.1-fold up-regulated and was shown to be responsible for the hypercalcemia associated with malignancy [13], but recent studies have revealed its growth-regulatory effects [14]. Next to its important regulatory effects on calcium, it is a growth factor for malignant cell lines of many tissues, including those of the bone, kidney and prostate [15], [16], [17]. Importantly, exaggerated Pthlh production is frequently observed in lung carcinoma cell lines and was found to be overexpressed in all types of lung carcinoma [18], [19]. In contrast, among the down-regulated genes several code for guanine nucleotide exchange factors (GEFs). In general GEFs stimulate the dissociation and exchange of bound GDP (guanosine diphosphate) for GTP (guanosine triphosphat) on Ras in response to upstream stimuli. Thus, binding of GTP activates RAS proteins. In adenocarcinoma RasGRP2 is −9.3-fold down-regulated and a similar −9.9-fold down-regulation was determined in a comparison with non-transgenic cells. Notably, RasGRP2 is targeted to the plasma membrane by a combination of N-terminal myristoylation and palmitoylation [20]. We found RasGRP3 −5.4-fold down-regulated in adenocarcinoma when compared with transgenic but otherwise unaltered cells and −6.9-fold down-regulated in a comparison with non-transgenic cells. Unlike the down-regulated GEFs, we found RasGRF1 (RAS protein-specific guanine nucleotide-releasing factor 1) 11.3-fold up-regulated in adenocarcinoma as compared with transgenic and 25.4-fold in the comparison adenocarcinoma vs non-transgenic. Specifically, RasGRF1 is a calcium-stimulated exchange factor for Ras and Rac. Additionally, RasGRF1 is a substrate of the tyrosine kinase Src and it seems that oligomerization of RasGRFs is required for its biological functions [21].

Surprisingly, the Ras GTPase-activating proteins (RasGAPs) are not regulated in lung adenocarcinoma. Although both the RasGEFs and RasGAPs proteins are needed to complete a full GTPase cycle and to allow for a switch between an inactive and active state of Ras, the activation state of these Ras proteins, but not the level of expression, might determine the cellular effects. Additionally, we found down-regulated Rho guanine nucleotide exchange factors such as ArhGEF15 (Rho guanine nucleotide exchange factor 15) (−9.4-fold adenocarcinoma vs transgenic/−10.7-fold adenocarcinoma vs non-transgenic), ArhGEF10 (Rho guanine nucleotide exchange factor 10) (−5.1-fold adenocarcinoma vs transgenic/−6.3-fold adenocarcinoma vs non-transgenic) and the Rho GTPase activating protein, ArhGAP20 (Rho GTPase activating protein 20) (−6.0-fold adenocarcinoma vs transgenic/−8.5-fold adenocarcinoma vs non-transgenic) to be repressed. Like Ras proteins, these Rho proteins are activated via extracellular signals that cause binding and hydrolysis of GTP and induction of downstream effector molecules [22].

Next to the members of the GEF family we observed regulation of molecules in the Wnt signalling pathway. Indeed, Wnt proteins comprise a family of secreted glycoproteins that play diverse roles in development, cell proliferation, cell polarity and cell fate determination [23], [24]. Binding of wnt molecules at frizzled transmembrane receptors stimulates an activation of the Wnt signal pathway. By activation of the canonical Wnt signal pathway the formation of the axin of mediated degradation complex is inhibited and cytosolic β-catenin is stabilized. As a consequence β-catenin enriches itself in the nucleus to interact with transcription factors of the Tcf/Lef family. We observed Wnt2 (wingless-related MMTV integration site 2) to be −13.8-fold repressed in cancer and -16.2-fold down regulated when compared to non-transgenic cells. Importantly, Wnt genes play a role as mediators of epithelial-mesenchymal interactions in the developing lung. It has been shown that Wnt2 is predominantly expressed in the mesenchyme during the lung development [25]. Evidence suggests that the Wnt signal pathway is required for the specification and differentiation of lung epithelial cells [26], [27] and plays a role in lung cancer [28], [29]. Nonetheless, alterations in the Wnt signal pathway in lung cancer are still unclear and are rather controversial. For example, it has been reported that immunohistochemical positive staining of β-catenin in the nucleus was observed in less than 10% of the primary non-small cell lung adenocarcinomas and was associated with better prognosis [30]. In contrast, reports suggest components of the Wnt signal pathway in metastatic lung cancer to be induced [27]. Moreover, in the non-small cell lung cancer cell line H292, β- catenin as well as further genes of the Wnt signal pathway were up-regulated [31]. Likewise, an up-regulation of Wnt5a was observed in lung carcinoma and discussed in connection with tumor progression [32]. Unlike our results, Wnt2 was reported to be increased in human non-small cell lung cancer tissues [33]. We also observed repression of Tcf21. This protein belongs to the family of basic helix-loop-helix transcription factors and was −17.7-fold down regulated in cancer compared to transgenic and −20.8-fold compared to non-transgenic. Tcf21 is known to forster differentiation of epithelial cells adjacent to mesenchymal and is a critical molecule for lung development [34]. In fact frequent promoter hypermethylation of Tcf21 was reported in primary lung adenocarcinomas [35].

Besides altered Wnt signaling molecules we found Notch pathway to be regulated as well. Notch 4 was −6.8-fold down regulated in adenocarcinoma compared to transgenic and −7.6-fold down regulated in the comparison adenocarcinoma versus non-transgenic. Specifically, Notch 4 (Notch gene homolog 4), a member of the transmembrane Notch family of receptors is expressed primarily in embryonic endothelium and in adult endothelium [36]. Upon binding of its ligand, Delta4, this receptor becomes activated. When activated, the intracellular subunit translocates into the nuclei and regulates transcription of many genes and regulates, among others, vascular endothelial cell growth factor (VEGF), NFκB and Hes-1 [37], [38]. Activation of Notch signalling plays important roles in development and maintenance of neoplastic phenotypes [39], [40], [41].

### Remodelling of cell adhesion

We found Chl1 (cell adhesion molecule with homology to L1CAM) a member of the L1 gene family of neural cell adhesion molecules as 20.4-fold up-regulated but up to 50 genes were down-regulated, e.g. Icam2 (intercellular adhesion molecule 2) (−9.4-fold), Pecam1 (platelet/endothelial cell adhesion molecule 1) (−5.4-fold), Sell (selectin, lymphocyte) (−8.6-fold), Cdh5 (cadherin 5) (−8.4-fold), Itga1 (integrin alpha 1) (−4.0-fold) and Itga8 (integrin alpha 8) (−16.1-fold) in adenocarcinoma vs transgenic cells. Specifically, Chl1 is a member of the immunoglobulin superfamily of neural cell adhesion molecules and it is highly expressed in the central and peripheral nervous systems [42], [43], but, L1 homologues have been described for other cell types of diverse origin as well, including endothelial cells, epithelial cells, reticular fibroblasts, and cells of lymphoid and myelomonocytic origin [44], [45], [46]. Several studies link L1 to motile processes involved in tumor cell extravasation and glioma dissemination in the brain [47]. Recent reports link L1 expression to melanoma metastasis [48] and ovarian and uterine carcinomas associated with poor clinical outcome [49] but, regulation of Chl1 in lung cancers is novel and may act as coreceptor for integrin and growth factors [50]. In this regard, Icam2, i.e. a member of the immunoglobulin supergene family of adhesion proteins that serves as the counter-receptor for leukocyte function-associated antigen-1 (LFA-1/or CD11a/CD18) is of considerable interest [51]. It is closely related to Icam1, but has only two Ig-like extracellular domains, as compared with the five Ig domains of Icam1 [52]. Icam2 cytoplasmic tail can interact with ezrin in vitro. Ezrin is a member of the ERM (ezrin, radixin, and moesin) family, which can function as a linker between the plasma membrane and the actin cytoskeleton [53]. Recently, it has been reported, that an increased Icam2 expression on cancer cells enhances the adhesion and reinforces the cytotoxic activity of immune cells such as NK cells, resulting in a reduction of metastasis [54]. We found Sell (L-selectin) to be regulated which codes for a lectin-binding protein and known to be expressed by leukocytes. This adhesion molecule mediates the initial attachment of leukocytes to endothelial cells, which allows leukocytes to roll along the venular wall [55], [56]. Lymphocytes and neutrophils exhibit a reversible loss of Sell expression after cellular activation that results from endoproteolytic release of the receptor from the cell surface [57]. Soluble Sell is functionally active and is present at high levels in human plasma. Decreased levels of L-selectin are observed in the serum of patients who develop adult respiratory distress syndrome [58]. It has been shown that Sell is able to transmit intracellular signals, including increased tyrosine phosphorylation and activation of MAP kinase in neutrophils [59]. There is increasing evidence for Pecam1 a cell adhesion molecule to interact with integrinα_v_β_3_ and plays a role in angiogenesis [60]. Moreover, endothelial cell tube formation depends on Pecam1 and cadherin 5 [61], which, in our study, were down-regulated. In this regard cadherin 5, a major cadherin in endothelial cells, is preferentially localized at interendothelial cell junctions [62]. Cadherins are organized in junctional structures, i.e. adherens junctions. In such junctions, cadherins are clustered and connected through their cytoplasmic domain with a complex network of cytoskeletal proteins. Through their homophilic interactions, they play a role in sorting cells of different lineages during embryogenesis, establishing cell polarity, as to maintain tissue morphology and cell differentiation [63], [64].

### Regulation of tight and gap junctions

We found Cldn5 (claudin 5) to be significantly repressed in lung adenocarcinoma. A functional relationship has been proposed for Cdh5 to control Cldn5 expression [65]. Specifically, Claudin 5 is a major claudin identified in normal endothelial cells [66] but is also expressed in type II alveolar epithelial cells [67]. It forms homooligomeres and is localized at the apical-tight junction region in both bronchi and bronchioles [68].

Furthermore, many reports suggest that adherens junctions (AJs) and tight junctions to be interconnected and that AJs influence tight junction organization. Regulation of claudin 5 expression by cadherin 5 may therefore reinforces such crosstalk between tight and adherens junctions to promote tumor growth and metastasis.

We found Cldn2 (13.4-fold) significantly up-regulated in adenocarcinoma. Cldn2 (claudin 2) has been shown to modify tumor invasion by the regulation of matrix metalloproteinases (MMPs). Both claudin 2 and claudin 5 are able to activate membrane-type 1-MMP-mediated pro-MMP-2 processing. Reports suggest members of the claudin-family to be modulated during oncogenic transformation and may therefore play an important role in influencing tumor progression and invasion [69], [70].

Besides significant regulation of tight junction coding genes we found the gap junction protein Gja5 (gap junction membrane channel protein alpha 5 or so called connexin 40) −7.3-fold down regulated in adenocarcinoma and −8.7-fold repressed when compared to non-transgenic cells. Gja5 is a member of the connexins and encodes a gap junction protein that is highly expressed in lung, vascular endothelium, and portions of the cardiac conduction system [71]. Recent studies confirmed Gja5/Cx40 gene expression in lung tissue [72] and that Gja5/Cx40 was expressed similarly in normal lung and small size lung tumors, but its expression decreased in large size tumors [73]. Notably, aberrant gap junctional intercellular communication is an important feature of malignant cells and allows neoplastic cells to escape the tissue-specific growth control [74], [75].

### Regulation of tyrosine kinase receptors and their ligands

Several studies have examined the role of growth factor receptors and their ligands in the activation of Ras. Therefore we searched for regulated growth factors and their receptors and observed 5 genes to be up-regulated ranging between 6.3-26.6-fold, e.g. Areg (amphiregulin), Ereg (epiregulin), and up to 70 genes down-regulated (−3.8 to −29.4-fold), e.g. Fgf7 (fibroblast growth factor 7), Fgfr4 (fibroblast growth factor receptor 4), Pdgfb (platelet-derived growth factor beta polypeptide)), Vegfd/Figf (c-fos induced growth factor) and Vegfr2/Kdr (vascular endothelial growth factor receptor- 2) when transgenic but morphologically unaltered lung tissue was compared with adenocarcinomas. Likewise, 12 genes were up-regulated between 7.4 to 598.1-fold and 71 genes were down-regulated (−4.1 to −30.4-fold) when compared to non-transgenic lung tissue. In particular, the Areg (amphiregulin) and Ereg (epiregulin), ligands of the epidermal growth factor receptor (Egfr) were highly significantly upregulated (up to 600-fold). It has been suggested that elevated expression of Areg and Ereg may play an important role in tumor growth and result in chronic Egfr stimulation followed by increased proliferation. Indeed, epiregulin is normaly repressed in human cells but reported to be extensively activated in tumors [76] but one study showed that activation of telomerase and subsequent induction of epiregulin are required for sustained cell proliferation [77]. Likewise, Areg transcript was identified in a variety of human tumors [78]. Importantly, recent evidence suggests Areg to inhibit apoptosis in non-small cell lung cancer cell lines [79]. Besides the significantly up-regulation of Areg and Ereg, we found fibroblast growth factor 7 −5.4-fold-down-regulated in adenocarcinoma and −6.4-fold down-regulated in adenocarcinoma vs non-transgenic cells. Additionally, Fgfr4 was −15.2-fold and −18.6-fold down-regulated in cancer. This monomeric receptor tyrosine kinase can induce angiogenic, mitogenic and differentiation responses in cells [80]. Dysregulation of this pathway was demonstrated in several tumors including lung cancer [81] but unlike our study, components of the FGF signalling pathway were reported to be up-regulated [82].

In this regard, we found Vegfd/Figf (c-fos induced growth factor) to be −28.7-fold down-regulated in adenocarcinoma as compared to transgenic and −30.3-fold down-regulated vs non-transgenic cells, while Vegfr2/Kdr (vascular endothelial growth factor receptor- 2) was −7.1-fold and −8.3-fold repressed, respectively. Specifically, Vegfd is a ligand for the Vegf receptor tyrosine kinases and activates the structurally similar type III receptor tyrosine kinase Vegfr3 [83]. Recently, it was shown that Vegfr2 mRNA level in peripheral blood was increased in NSCLC patients when compared with healthy individuals [84]. However, this finding is not supported by our results. Note, in the context of angiogenesis we found angiopoietin 1 (Angpt1) and tyrosine kinase with immunoglobulin and epidermal growth factor homology domains (TIE1) also down regulated in adenocarcinoma. Both molecules are involved in vascular homeostasis, vascular integrity and angiogenesis.

A further down regulated gene was PdgfB (platelet-derived growth factor beta polypeptide). It was −4.0-fold repressed in adenocarcinoma compared with transgenic but otherwise unaltered cells and −4.6-fold down regulated in adenocarcinoma compared with non-transgenic. Importantly, the B chain of Pdgf is the cellular homologue of the v-sis oncogene, which is known to cause simian sarcoma [85], [86] and a homodimer composed of two B chains is a potent mitogen for a variety of cells [87]. Although the contribution of PdgfB in tumour genesis is not fully clarified, the co-expression of PdgfB and its receptors has been described in many tumours, and autocrine PdgfB stimulation in these tumours has been suggested [88], [89]. It was shown that exon 1 of PdgfB was deleted and replaced by a variable segment of collagene 1A1 gene [90] thereby activating the v-sis oncogene in experimental models [91].

### Pertubation in Lipid metabolism

Numerous investigations have demonstrated changes in lipid metabolism in cancer patients, as well as aberrant lipid utilization by tumor cells [92], [93]. Therefore we examined genes involved in lipid metabolism and found 11 genes in adenocarcinoma to be regulated ranging from −3.6 to −23.1-fold. In particular Hpgd (15- hydroxyprostaglandin dehydrogenase) was significantly −23.1-fold down regulated in adenocarcinoma vs transgenic and −26.6-fold down regulated in adenocarcinoma vs non-transgenic cells. Note, this dehydrogenase is highly expressed in the lung and a key catabolic enzyme to control the biological activity of prostaglandins [94], [95], [96], by converting PGE_2_ into the less active metabolite 15-hydroxy-PGE_2_. In addition to the prostaglandins, the −ω - 6 hydroxy fatty acids, 15HETE; 5,15-di-HETE; 8,15-diHETE [97] and lipoxin A, [98] are also substrates for Hpgd. A small but significant increase in PGE_2_ in the lungs of Hpgd knock out animals was reported [99].

Next, we found Pitpnc1 (phosphatidylinositol transfer protein, cytoplasmic, 1) −12.0-fold down regulated in adenocarcinoma vs transgenic and −14.2-fold down regulated compared with non-transgenic. The Pitpnc1 belongs to a family of phosphatidylinositol transfer proteins and recent studies suggest Pitp to be required to maintain levels of PI(4,5)P_2_ during G-protein coupled Plcβ signalling [100].

The Pltp (phospholipid transfer protein) was also −7.5-fold down regulated in adenocarcinoma vs transgenic and −8.6-fold as compared to non-transgenic cells. This transfer protein is a member of the lipid transfer/lipopolysaccharide binding protein gene family, that play a major role in the metabolism of HDL, vitamin E and lipopolysaccharide [101]. Pltp facilitates cholesterol efflux from cells and, enhances the transfer of surface remnants from triglyceride-rich lipoproteins to HDL during lipolysis [102], [103].

### Regulation of acyltransferases

Next to lipid metabolism we examined genes involved in carbohydrate metabolism and found several acetyltransferases to be regulated.

For example, Oact1 (O-acetyltransferase (membrane bound) domain containing 1) was 6.4-fold up-regulated in adenocarcinoma when compared with transgenic but healthy cells. Oact1 belongs to the family of membrane-bound O-acyltransferases (MBOATs) which are important for membrane phospholipids biosynthesis [104]. Oact1 contributes to the turnover of phospholipids and is involved in catalysis of membrane biogenesis [105]. We observed altered expression of genes coding for cell surface glycolipids in adenocarcinoma. Several were already up-regulated in dysplasia, for example St8sia6 (St8 alpha-n-acetyl-neuraminide alpha-2,8-sialyltransferase 6), Orm1 (orosomucoid 1) and Gpc6 (glypican 6). In adenocarcinomas, the expressions of these molecules were also increased. In contrast, we found chondroitin sulfate proteoglycan 4, UDP-N-acetyl-alpha-D-galactosamine: polypeptide N-acetylgalactosaminyltransferase-like 4, 1-acylglycerol-3-phosphate O-acyltransferase 4, ST8 alpha-N-acetyl-neuraminide alpha-2,8-sialyltransferase 2, glycoprotein Ib/beta polypeptide, lecithin-retinol acyltransferase, glycoprotein m6a, carbohydrate sulfotransferase 1 were up to -20-fold down regulated.

Specifically, chondroitin sulfate proteoglycan 4, a cell surface proteoglycan is known to regulated in the adhesion, migration and invasion of tumor cells such as human melanoma and infantile acute myeloid leukemia [106]. The second down regulated glycoprotein was glycoprotein Ib/beta polypeptide, a platelet surface membrane glycoprotein that functions as a receptor for von Willebrand factor [107] and was shown to affect receptor endocytosis and recycling [108]. We found glycoprotein m6a −19.5-fold down regulated in adenocarcinomas. Additionally, 1-acylglycerol-3-phosphate O-acyltransferase 4 also known as lysophosphatidic acid acyltransferase was −5.0-fold down regulated in adenocarcinomas. This enzyme is a crucial for synthesis of glycerolipids as well as triacylglylcerol biosynthesis. It catalyses the acylation of lysophosphatidic acid (LPA) to form phosphatidic acid (PA), the precursor of all glycerolipids [109].

We also observed the ST8 alpha-N-acetyl-neuraminide alpha-2,8-sialyltransferase 2 to be nearly 6-fold down regulated in adenocarcinoma. This transferase catalyzes the polycondensation of alpha-2,8-linked sialic acids to polysialic acid (PSA) and the attachment of polysialic acid to the carrier molecule e.g. Ncam. Polysialic acids are implicated in numerous normal and pathologic processes, including tumor metastasis [110]. A further down regulated transferase was lecithin-retinol acyltransferase (phosphatidylcholine-retinol-O-acyltransferase). We found Lrat up to -13-fold down regulated in adenocarcinoma. This enzyme is essential in the processing of vitamin A and is responsible for conversion of all-trans-retinol into retinyl esters [111].

### Regulation of embryonic development programs

Cancer cells utilise embryonic programms for instance by producing blocking factors to inhibit the immune system from attacking them. Here, we focused on genes coding for molecules involved in cellular development and found 9 genes up-regulated ranging between 5.9 to 26.6-fold and 56 genes down-regulated ranging from −3.6-fold to −29.4-fold in adenocarcinoma.

This included Fst (follistatin) which we found 6.3-fold up-regulated. This single chain autocrine glycoprotein [112] is a functional antagonist of several TGF-β proteins such as activin A [113], [114]. Follistatin is involved in the development of the embryo and has inhibitory action on bone morphogenic proteins (BMPs) [115], [116]. Notably, Bmp6 (bone morphogenetic protein 6) was significantly −29.4-fold down regulated in adenocarcinoma when compared with transgenic cells and −35.2-fold down regulated in adenocarcinoma versus non-transgenic cells. Bmps are part of the transforming growth factor (TGF)-β super-family of secreted signaling molecules [117]. Growing evidence suggest deregulation of follistatin and activin to contribute to cancer development and metastasis [118], [119], [120].

Furthermore some of the homeobox genes, e.g. Hoxa5, Hoxb5, Meis1 and Mrg1 were up to 9-fold repressed in adenocarcinomas. During morphogenesis Hox factors specify body segments [121] and are expressed in defined and often overlapping domains along the anterior-posterior axis. It has been shown that Hox proteins work together with many other transcription factors in a combinatorial fashion [122]. Importantly, Hoxa5 and Hoxb5 play a role in morphogenesis and early differentiation of lung [123], [124] and the extent of transcript expression correlates with the level of methylation [125]. Promoter methylation could be involved in tissue specific silencing of these genes and had been reported for the Hoxa5 gene in lung cancer [126], in human breast tumors [127], in myeloid and lymphoid malignancy [128] and for the Hoxb5 gene in ovarian carcinomas [129] where the gene undergoes de novo methylation. Additionally, it was shown that Hoxa5 up-regulates the tumor suppressor gene p53 [127]. We observed regulation of Meis 1 and Mrg1 in adenocarcinomas. These encode homeobox proteins belonging to the TALE (‘three amino acid loop extension’) family of homeodomain-containing proteins and were first identified, as a common viral integration site in myeloid leukemic cells of BXH-2 mice [130]. Meis1, Mrg1 and other members of the TALE family serve as Hox cofactors by altering HOX-DNA binding specificity, to increase DNA binding affinity thereby augmenting their transcriptional activity [131]. Their coordinate regulation in adenocarcinomas as reported herein has also been shown in ovarian cancers [132] and in the IMR32 neuroblastoma cell line [133].

Next to the homeobox genes we found members of the transcription factors belonging to the SRY-related HMG (High Mobility Group) box superfamily down regulated in adenocarcinoma e.g. Sox7, Sox11 and Sox13 were repressed by −4.9 to −80.6 and −4.3-fold in adenocarcinoma compared with transgenic and −5.6 to −101.0-fold in adenocarcinoma versus non-transgenic. They are involved in a number of developmental processes and are expressed in a cell-specific way [134]. Deregulations of Sox genes are associated with a large number of tumour types in vivo [135], [136], [137].

Specifically, Sox7 plays a critical role in endoderm differentiation. The silencing of Sox7 was shown to be inhibited by Gata-4 and Gata-6, which are transcription factors as well [138]. Sox7 mRNA is highly expressed in adult lung but down-regulated in lung cancer [139]. In prostate cancer tumor-specific promoter hypermethylation of Sox7 was reported [140]. Additionally, we observed regulation of Sox11

Next, we observed repression of Gata nuclear transcription factors. GATA-6, a member of the GATA family of zinc finger proteins, is the only family member known to be expressed in the epithelial cells of the developing airway epithelium [141]. It has been reported that a deficiency of Fog2 (Friend of Gata 2), causes loss of the accessory lobe and anterior right medial lobe of the mouse lung with relatively good preservation of other structures [142] and that a Fog2-Gata4 interaction is critical for the development of normal pulmonary lobar structure [143]. Here we evidence Gata-2 and Gata-3 to be up to −12.7-fold down regulated in adenocarcinoma. These factors have been shown to play essential roles in development, such as for hematopoietic progenitor cell function [144].

### Regulation of tumor suppressors

Expression of the tumor suppressors mutated in colorectal cancers (Mcc), hairy/enhancer-of-split related with YRPW motif 1 (Hey1), FAT tumor suppressor homolog 3 (Fat3), armadillo repeat containing, X-linked 1 (Armcx1) and reversion-inducing-cysteine-rich protein with kazal motifs (Reck) was significantly up to −14.0-fold repressed in adenocarcinomas.

Notably, the Mcc tumor suppressor gene was found to be mutated in several colorectal tumors [145]. This membrane-associated cytoplasmic phosphoprotein blocks cell cycle progression from the G_0_/G_1_ to the S-phase [146] and therefore plays a role in cell cycle progression. Decreased Mcc mRNA levels were shown in neoplastic mouse lung tissue and cell lines [147]. Importantly, loss of heterozygosity at the Apc/Mcc gene cluster on chromosome 5q21 is frequent in non-small lung cancer [148]. We also observed repression of Fat3, a planar cell polarity signalling molecule in the Drosophila wing and a member of the cadherin superfamily [149].

Additionally, Hey1 was up to −10.0-fold repressed in adenocarcinoma. This basic helix–loop–helix transcription family plays a role in blood vessel formation and is involved in proliferation, migration, and network formation of endothelial cells [150]. It is induced during endothelial cell tube formation and down-regulates vascular endothelial growth factor (VEGF) receptor 2 in endothelial cells [151]. Recently, it was shown that Hey1 activates p53 through repression of Mdm2 transcription [152].

### Quantitative real-time PCR

**Figure 9:**
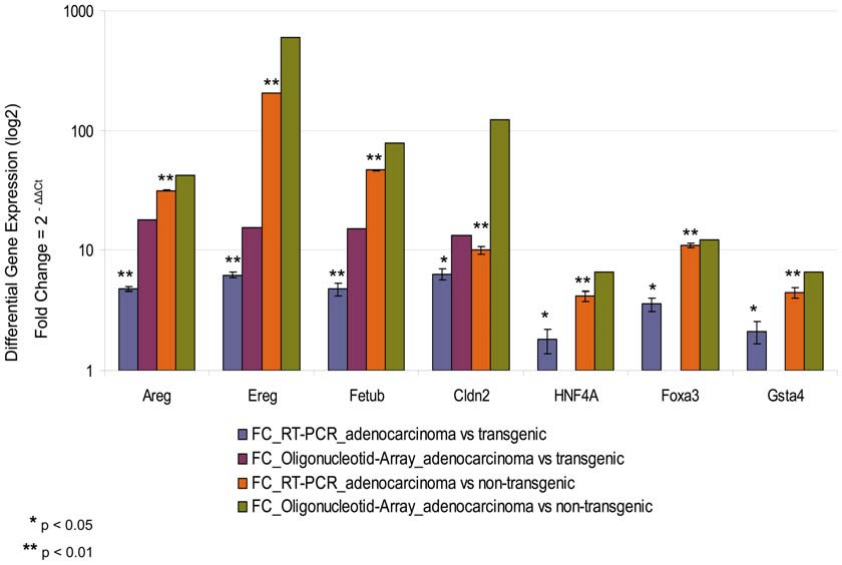
Comparison quantitative RT-PCR and Oligonucleotid-Array. Summary of differential expression of the seven genes verified by quantitative RT-PCR in comparison with Oligonucleotid-Array analysis. The data were analyzed statistically using Student's t-test (*p<0.05; **p<0.01). Error bars indicate standard deviation of five samples and two independent assays for each gene.

Expression of 7 genes in adenocarcinoma, transgenic and non-transgenic samples was examined by quantitative real-time PCR using TaqMan Technology. RT-PCR confirmed that amphiregulin (Areg), epiregulin (Ereg), fetuin beta (Fetub), claudin 2 (Cldn2), hepatic nuclear factor 4, alpha (Hnf4α), glutathione S-transferase, alpha 4 (Gsta4) and forkhead box A3 (Foxa3) were up-regulated in adenocarcinoma (Supplementary Figure S3 and S4). The expression data generated by the oligonucleotid array and RT-PCR agreed well, therefore supporting the reliability of the array analysis (Figure 9).

### Immunohistochemistry

**Figure 10:**
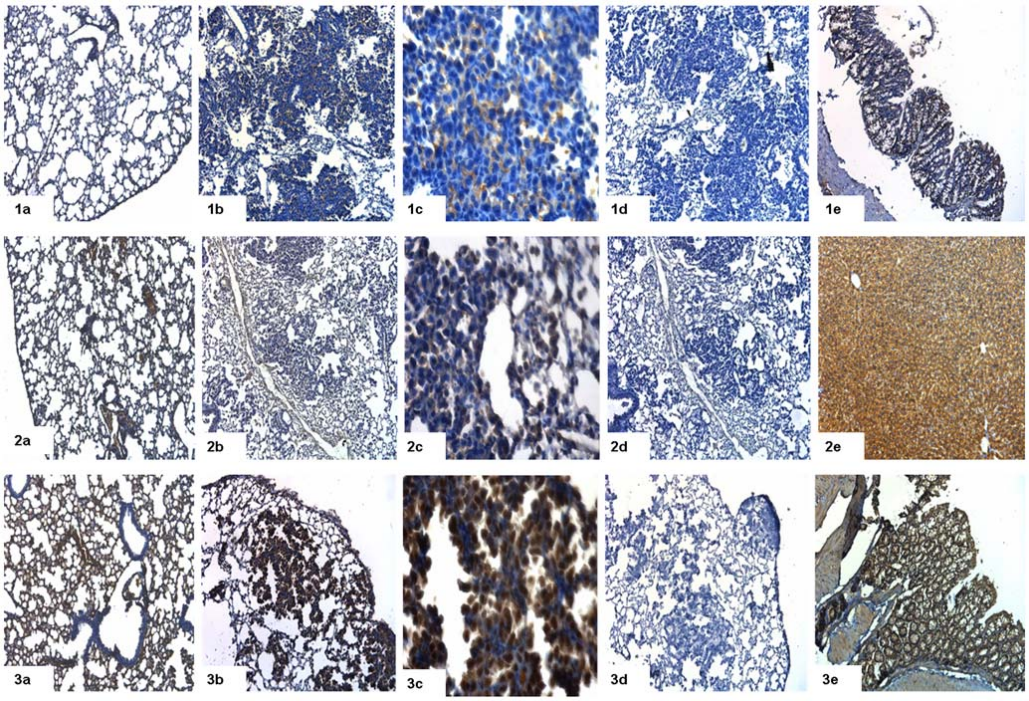
Immunohistochemical staining for adenocarcinoma (Cldn2, Fetub, Fst). (a) Immunohistochemical staining of control lung tissue, (b) lung cancer at 10× magnification and (c) at 40× magnification (d) lung cancer in the presence of primary antibody, after preincubation with blocking peptide and (e) positive control. 1 = claudin 2 (Cldn2), 2 = fetuin beta (Fetub), 3 = follistatin (Fst).

**Figure 11:**
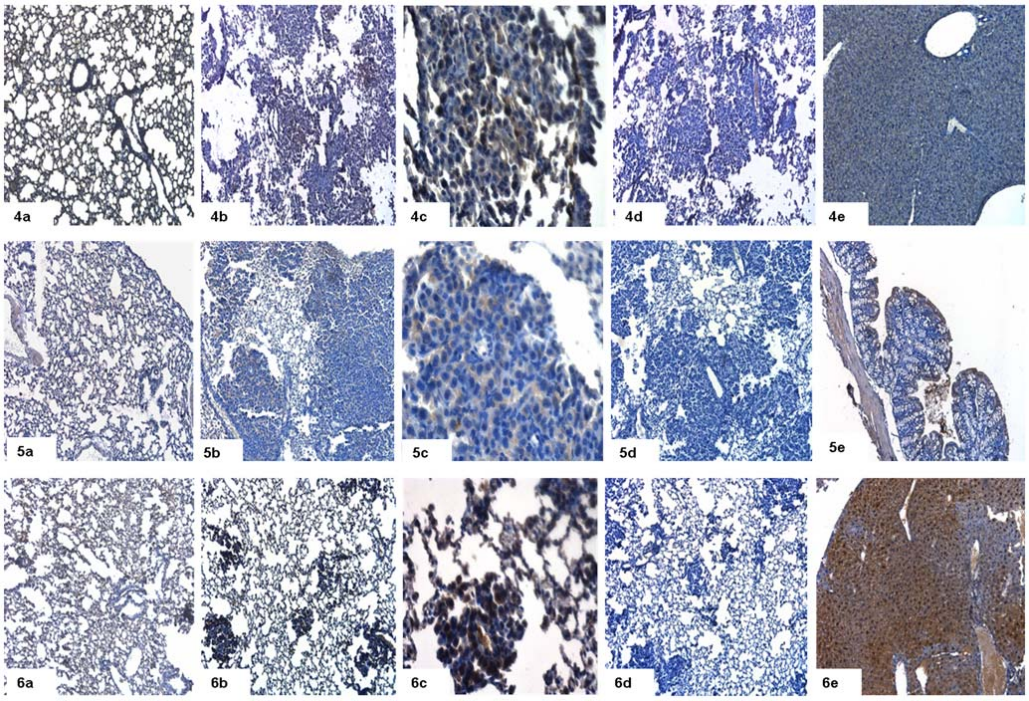
Immunohistochemical staining for adenocarcinoma (Orm1, Mcc, RasGRF1). (a) Immunohistochemical staining of control lung tissue, (b) lung cancer at 10× magnification and (c) at 40× magnification (d) lung cancer in the presence of primary antibody, after preincubation with blocking peptide and (e) positive control. 4 = orosomucoid 1 (Orm1), 5 = mutated in colorectal cancers (Mcc), 6 = guanine nucleotide-releasing factor 1 (RasGRF1).

**Figure 12:**
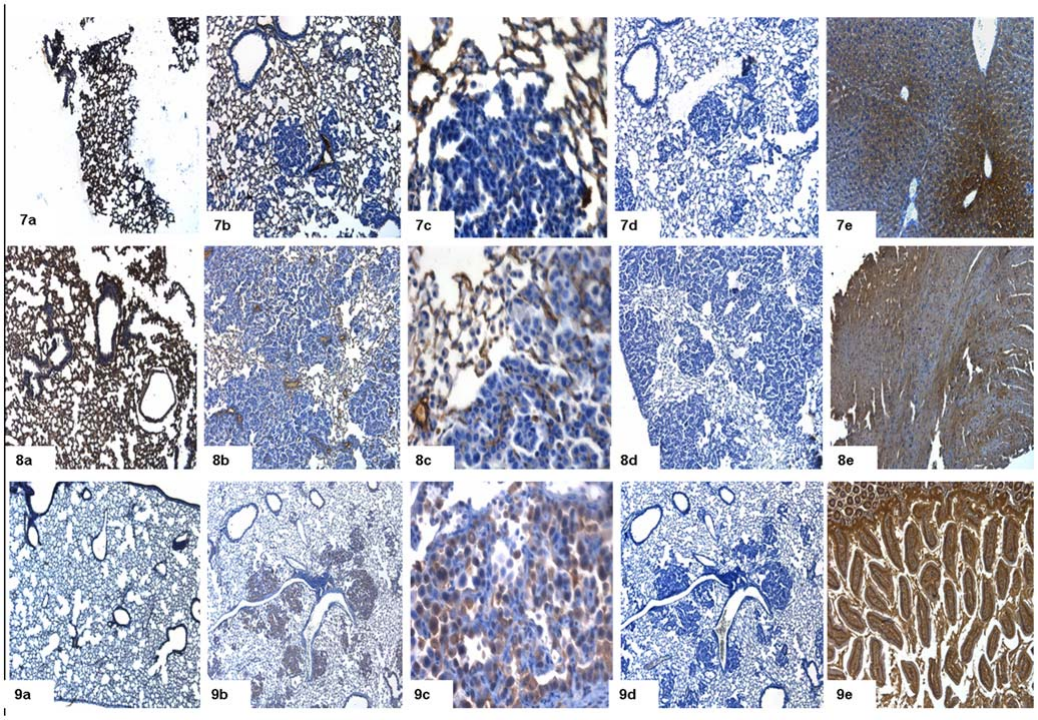
Immunohistochemical staining for adenocarcinoma (Reck, Cdh5, S100A14). (a) Immunohistochemical staining of control lung tissue, (b) lung cancer at 10× magnification and (c) at 40× magnification (d) lung cancer in the presence of primary antibody, after preincubation with blocking peptide and (e) positive control. 7 = reversion-inducing-cysteine-rich protein with kazal motifs (Reck), 8 = cadherine 5 (Cdh5) and 9 = S100 calcium binding protein A14 (S100A14).

**Figure 13:**
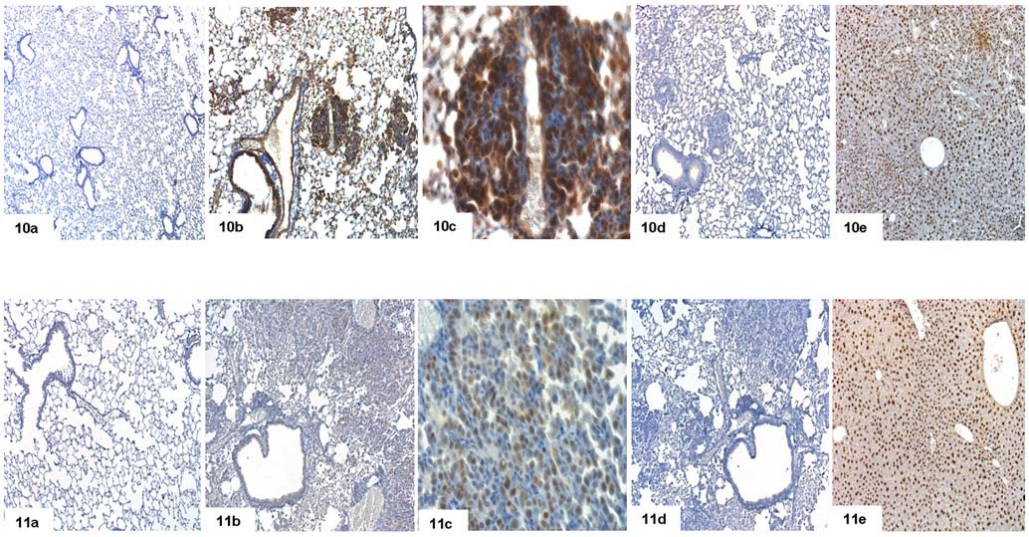
Hnf4α expression in dysplasia and adenocarcinoma. Hnf4α staining. 10 = dysplasia, 11 = adenocarcinoma (a) Immunohistochemical staining of control lung tissue, (b) lung cancer at 10× magnification and (c) at 40× magnification (d) lung cancer in the presence of primary Hnf4α antibody, after preincubation with blocking peptide and (e) positive control.

To further validate gene expression data we performed immunohistochemical staining for some of the differentially expressed genes (Figure 10, 11, 12). Immunohistochemistry using antibodies targeted against Cldn2 (claudin 2), Fetub (fetuin beta), Fst (follistatin), Orm1 (orosomucoid 1), S100a14 (S100 calcium binding protein A14), Mcc (mutated in colorectal cancers), RasGRF1 (guanine nucleotide-releasing factor 1), Reck (reversion-inducing-cysteine-rich protein with kazal) and Cdh5 (cadherine 5) confirmed the array data (Figure 10, 11 and 12). Notably, Cldn2, Fetub, Fst, Orm1, S100a14 and RasGRF1 were primarily detected in the cytoplasm of bronchial normal cells and tumor cells. Overall, tumor cells showed higher levels of protein expression than histologically normal bronchial epithelium. In the case of Fst and RasGRF1 strong cytoplasmic and nuclear staining was observed, where as the expression of Cldn2 was restricted to the cytoplasm and patchy throughout neighboring cancer cells. Importantly, in the case of Cdh5 and the tumor suppressors Mcc and Reck tumor cells are largely unstained while the underlying stroma and the control lung tissue was positive. In Figure 13 expression of Hnf4α in dysplasia (10) and cancer (11) is depicted. Consistent with Hnf4α mRNA levels the Hnf4α protein levels were elevated in dysplasia as compared to adenocarcinomas.

## Discussion

Cancer genomics provides valuable insight into molecular abnormalities associated with the different stages of disease, i.e. from low and high grade dysplasia to malignant transformation. Here we report a comprehensive lung cancer genomic study in a transgenic mouse model that recapitulates distinct genetic events associated with adenocarcinomas. Distinguishing between different stages of disease provides important information for translational research both from a diagnostic point of view and with regards to novel therapeutic interventions.

**Figure 14:**
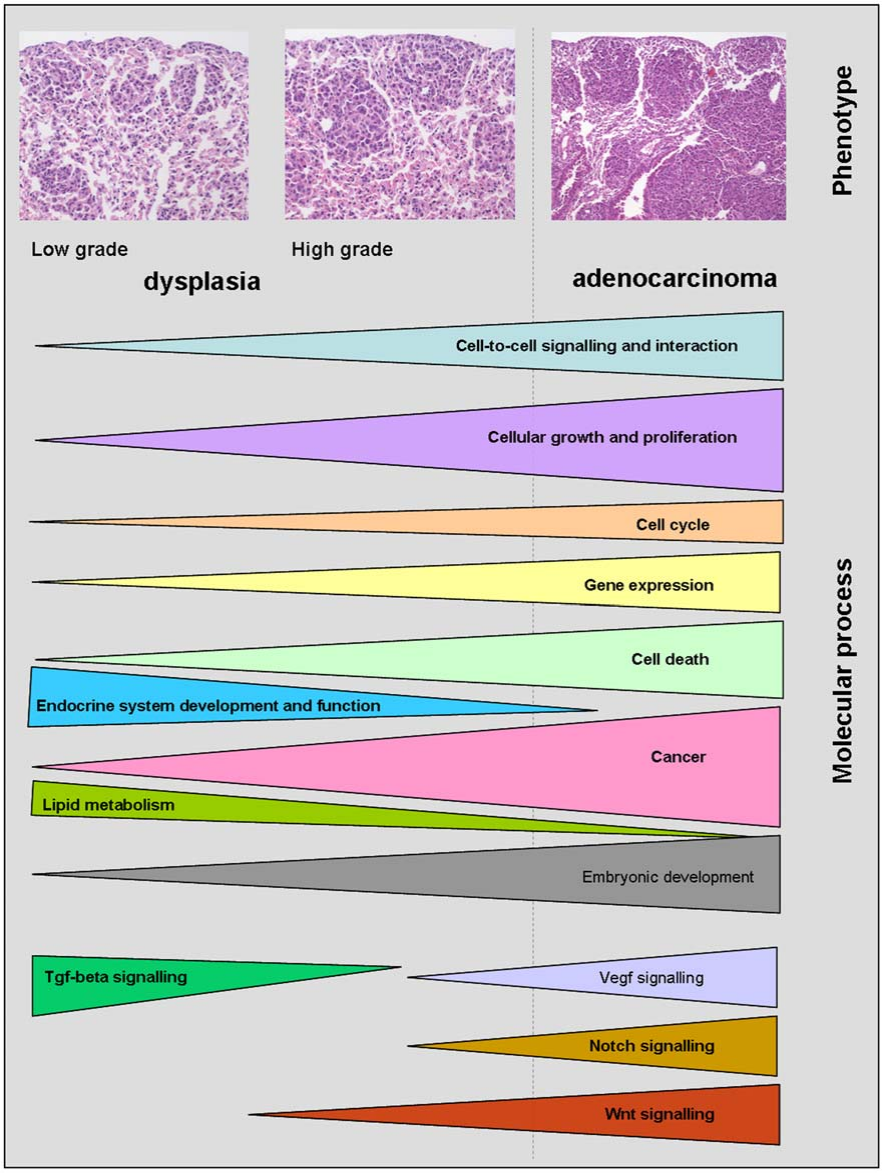
Schematic model of the lung tumor progression. Schematic model of the lung tumor progression based on molecular analyses of various grades of lung cancer. Shown are changes in molecular processes whereby the number of regulated genes and not the direction of gene expression were chosen.

Notably, lung cancer is a multistage process to result in perturbations of normal cellular regulatory mechanisms that ultimately leads to unchecked proliferation, infiltrative growth and metastasis. By use of whole genome arrays we were able to probe comprehensively for altered transcript expression associated with either transgenicity or dysplasia or cancer. Through advanced data analysis we could filter “genomic noise” and thus searched for regulatory networks in lung adenocarcinomas. Based on our recent study on dysplasia of respiratory epithelium [8] we suggest a sequence of events, i.e. nuclear atypia with low and high grade dysplasia followed by malignant transformation in fast growing highly undifferentiated tumor. Through an application of various bioinformatics tools networks could be constructed that appear to be hallmarks at distinct phases of tumor development therefore enabling fingerprinting of genes associated with pathological phenotypes. Indeed, the microarray study provided a wealth of information that allowed for hypothesis generation to earmark the switch from dysplasia to malignant transformation and the discovery of molecular events in lung cancer induced by exaggerated c-Raf activity (Figure 14).

As denoted by many investigators perturbation of signaling pathways may result in disruption of cell cycle regulation and of cell death. Here we identified regulatory gene networks associated with adenocarcinomas in a transgenic mouse model that already allowed us to asses precancerous stages of lung cancer induced by c-Raf. Particularly, the RAS proto-oncogen family (K-ras, H-ras, N-ras) codes for plasma membrane proteins. A mutation in the RAS gene inadequately extendes Ras-Raf-MAPK signaling to forster cell division. Approximately 90% of all ras mutations affect the K-ras gene, of which 85% occur in codon 12. K-ras mutations occur in particularly high frequency in adenocarcinoma (20–30%) but sequencing of k-ras and c-raf of our transgenic tumor model did not evidence any sequence alteration (data not shown). We therefore describe a tumor model where amplification of a aberrantly active c-Raf protein induced lung cancer. Indeed, undue activity of c-Raf in human lung cancer has been reported [10], [11].

We combined the power of microdissection with genome wide expression profiling. Because of the considerable heterogeneity of cells in and around tumor sites microdissection allowed us to harvest pure cell populations. Consequently, contamination of the investigated tumor cells by normal epithelial cells, blood vessels and cancer necroses that would otherwise result in misleading information can be avoided [153]. Based on our recent report on dysplasia and the findings of the present study we put forward a model for successive stages of disease, with dysplasia being at the edge of malignant transformation. We identified processes specifically regulated in dysplasia that is a facultative cancer. This included altered cell-to-cell signaling and cell-to-cell interaction, perturbations in lipid metabolism, glycosylation, an array of post translational modifications as well as undue regulation of development and cellular movement programs [8].

We then compared regulation of genes in dysplasia with tumors and identified up-regulated genes in adenocarcinoma, some of which were already over expressed in dysplasia. These genes coded particularly for components of receptor tyrosine kinases and biological onthologies assigned to cell-to-cell signalling, lipid metabolism, development and cancer. In dysplasia, however and unlike adenocarcinomas, we could not evidence perturbations in other signalling pathways such as MAPK, JAK/STAT, Wnt, PI3K/AKT and Notch. Notably, many genes were repressed in adenocarcinomas and could be grouped into the GO terms cell-to-cell signaling, cell adhesion and metabolism, regulation of development as well as repression of tumor suppressor genes. Indeed, many growth factor receptors mediate their cellular effects by intrinsic tyrosine kinase activity, which in turn, may phosphorylate other effector molecules involved in mitogenesis. A number of transforming oncogene products have growth factor or growth factor receptor-like activities that work via a tyrosine kinase-activating mechanism. We found members of vascular endothelial growth factor receptor family deregulated, such as Vegfr and Vegfd. Vegf is a key mediator of tumor angiogenesis [154] and inhibition of Vegf is a targeted approach in cancer therapy. Specifically, bevacizumab, a recombinant anti-vascular endothelial growth factor antibody, is in clinical development in the therapy of NSCLC. It was shown that addition of bevacizumab to chemotherapy (carboplatin/pacilitaxel) significantly improved overall survival, as well as progression-free survival and response rates [155]. However, our findings imply that in advanced tumor stages of adenocarcinoma members of the Vegf family are down regulated. This agrees well with the clinical observation of poor success of antiangiogenic therapies in advanced stages of disease [156]. In our tumor model aberrant expression of other members of the mitogen-activated protein kinase (MAPK) pathway was observed as well. As the MAPK pathway mediates a variety of responses to extracellular stimuli, dysregulation of some of its components in tumor cells was of no surprise to us and has been denoted by other investigators [157]. Furthermore, the observation that post-translational modifications, such as farnesylation are required for membrane localization and activation of Ras, has led to an interest in developing Ras inhibitors for varoius tumors, including leukemias, non small cell lung cancer, prostate, breast, pancreatic, and colorectal cancers by inhibiting farnesyltransferases (FTIs). Although inhibition of K-ras activity through inhibitors of farnesyltransferase could represent an important strategy to block cell proliferation, clinical results obtained to date have been disappointing [158]. Thus, the focus of development of a more viable approach to inhibit the MAPK pathway has turned to Raf and MEK. Sorafenib (BAY43-9006) is an oral, dual inhibitor of Raf and vascular endothelial growth factor receptor (VEGFR) and was initially developed as a specific inhibitor of the serine/threonine RAF kinase, in particular c-Raf (also known as Raf-1) and b-Raf [159]. The molecule has demonstrated preclinical antineoplastic activity against a wide spectrum of human cancers [160]–[164] eventhough some investigator suggests highly selective Raf-inhibitors to display more efficacy [165]. Indeed, XL-281 and PLX-4032 are oral inhibitors with high selectivity against Raf and are in phase 1 clinical trials.

A further important finding of our study was the discovery of regulated genes coding for cell adhesion. Notably, these molecules are already discussed in association with metastasis as shown for cadherin. Studies on the pathophysiology of metastasis of tumor cells suggest that dysregulation of adhesion molecules for the detachment of tumor cells from their primary cells are necessary but require a complex cascade of adhesive interactions between tumor cells and tissues. In the present study a variety of cell adhesion molecules had changed expression in advanced cancer growth that are involved in tight- and gap junctions such as the claudins and gap junction protein 5. Notably, claudin 2 was selectively expressed in lung adenocarcinomas, whereas claudin 5 expression was strongly repressed in lung tumors. Tight junction proteins are integral membrane proteins, which interact with the proteins of the neighboring epithelial or endothelial cell. They are essential for the maintenance of cell polarity. Already in some tumors, such as in breast cancer and gastric cancer repression of tight junction proteins was observed [166]. Dysfunction of these proteins leads to the disruption of the tight junction, a process that may play a major role in the loss of cohesion and poor differentiation of tumor cell [167]. Moreover, by the loss of tight junctions the orderly structure of tissue will no longer be maintained and tumor cells detach more easily from the tumor and invade surrounding tissues [166]. Therefore, alteration in tight junctional communications represents an important molecular mechanism in lung tumor progression.

Furthermore, genes involved in glycosylation of phospholipids were significantly altered. Indeed, cell surface proteoglycans are important in modulating cell adhesion and motility. Changes in glycosylation patterns during transformation of normal cells into cancer cells have been reported as to enable cancer cells to improve cell to cell interaction and adhesion to extracellular matrices.

Another finding of our study was the remarkable regulation of hox genes in adenocarcinoma, for example Hoxa5, Hoxb5, Meis1 and Meis2. It is known that the hox genes act in early development and code for transcriptional regulators that can turn genes on or off. Regulation of developmental programs in malignancy is the subject of intense research. Aditionally, we observed repression of several tumor suppressors. Loss of tumor suppressor gene function is germain to cancers.

### Gene regulatory networks

**Figure 15:**
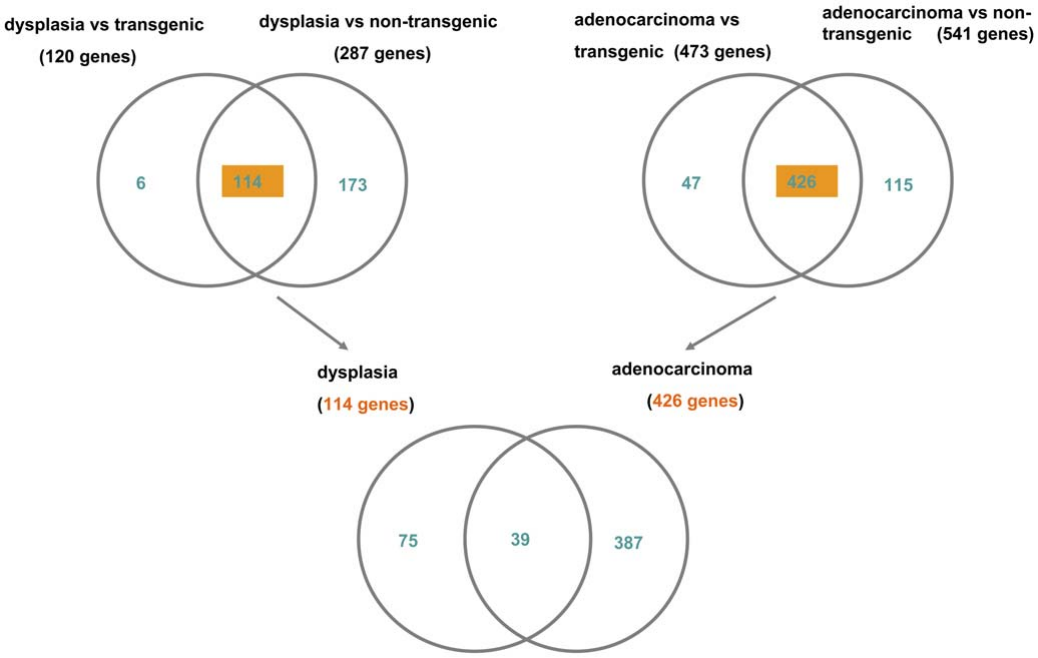
Venn diagram for the intersection of significantly differential expressed genes in dysplasia and adenocarcinoma. Comparison of tumor and transgenic unaltered lung tissue with non-transgenic samples (426 genes) and comparison of dysplasia versus transgenic and non-transgenic (114 genes).

We were particularly interested in identifying regulatory networks associated with the switch from dysplasia to malignant transformation. Based on bioinformatics analysis 75 genes were regulated exclusively in dysplasia (Supplementary Figure S5 and Supplementary Text S1), where as 387 genes could be attributed to adenocarcinoma of which 39 genes were regulated in common (Figure 15).

### Common networks in dysplasia and cancer

**Figure 16:**
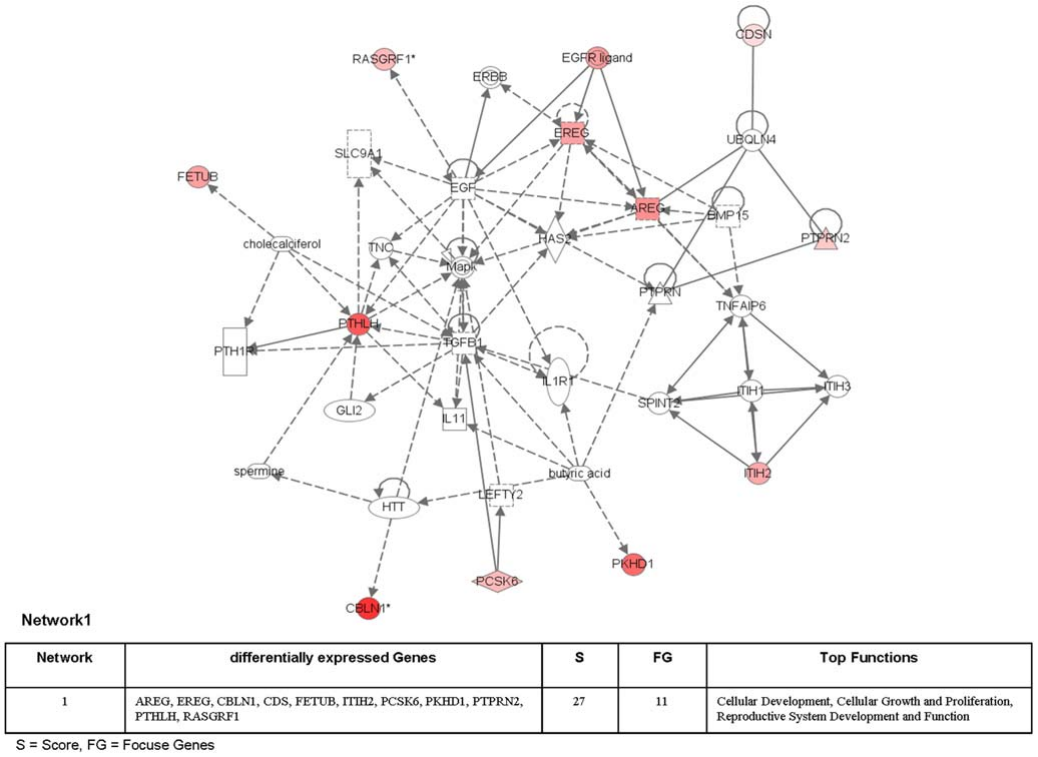
Ingenuity networks: common regulated genes in dysplasia and adenocarcinoma. Ingenuity networks generated by mapping the focus genes that were common expressed between dysplasia and adenocarcinoma.

**Network 1** (Figure 16) shows the common regulated genes in dysplasia and adenocarcinoma. The central components in this network are Egf, Mapk and Tgf-β eventhough they are themselves not regulated many of their downstream targets are. Notably, Areg (amphiregulin) and Ereg (epiregulin) are ligands of the EGF receptor tyrosin kinases and are members of the ERBB family including EGFR (ERBB1) to function as activators. The EGFR pathway stimulates cell proliferation and motility and overexpression of EGFR–ligands was observed in many kinds of cancer including lung, breast and prostate cancer. Furthermore, RasGRF1 (RAS protein-specific guanine nucleotide-releasing factor 1) is a direct target of EGF. RasGFR1 enhances the release of GDP that is required for Ras activation. There is cross-talk between the EGFR and MAPK signaltransduction pathways.

In this network Pthlh (parathyroid hormone-like hormone) is a further central component and is highly up-regulated. Based on the chick chorioallantoic membrane angiogenesis and mouse tumor angiogenesis assays it was postulated that Pthlh is a Pka dependent angiogenesis inhibitor in vivo [168]. In this context Fetuin B (Fetub) was identified as a downstream target of Pthlh. Notably, up-regulated Fetub was reported for several tumors [169].

A further target molecule in this network is Itih2 (inter-alpha-trypsin inhibitors heavy chain2). This protein acts as plasma serine protease inhibitor and stabilized extracellularmatrix proteins. Down regulation of ITIH proteins and Itih2 in particular has been reported in different types of cancer including breast and lung cancer [170].

Furthermore, Pcsk6 or PACE4 Paired Basic Amino Acid Cleaving Enzyme is a proprotein convertase that mediates the production of peptidic mitogens. Regulation of this gene was reported for non-small cell lung cancers. Maybe it is involved in autocrine production of mitogens contributing to unchecked proliferation of tumor cells [171].

Additional members of this network are Ptprn2 (protein tyrosine phosphatase, receptor type, N polypeptide 2) and Pkhd1 (polycystic kidney and hepatic disease 1), which act i.e. as signaling molecule involved in cell growth, differentiation, mitotic cycle, and oncogenic transformation. The Ptprn2 locus is reported to be hypermethylated in lung cancer [172] while Pkhd1 (polycystic kidney and hepatic disease 1) is associated with polycystic kidney and hepatic disease. In recent works it was shown that Hnf1β is required for the activation of the Pkhd1 promoter [173]. Finally, Cdsn (Cornedesmosin) is downstream of Ubqln4 (ubiquilin 4) a connexin43-interacting protein. Cds encodes for intracellular structures participating in desquamation. It is normally expressed in skin cells and until now regulation of this gene was not associated with tumors.

### Gene networks exclusively regulated in adenocarcinoma

**Figure 17:**
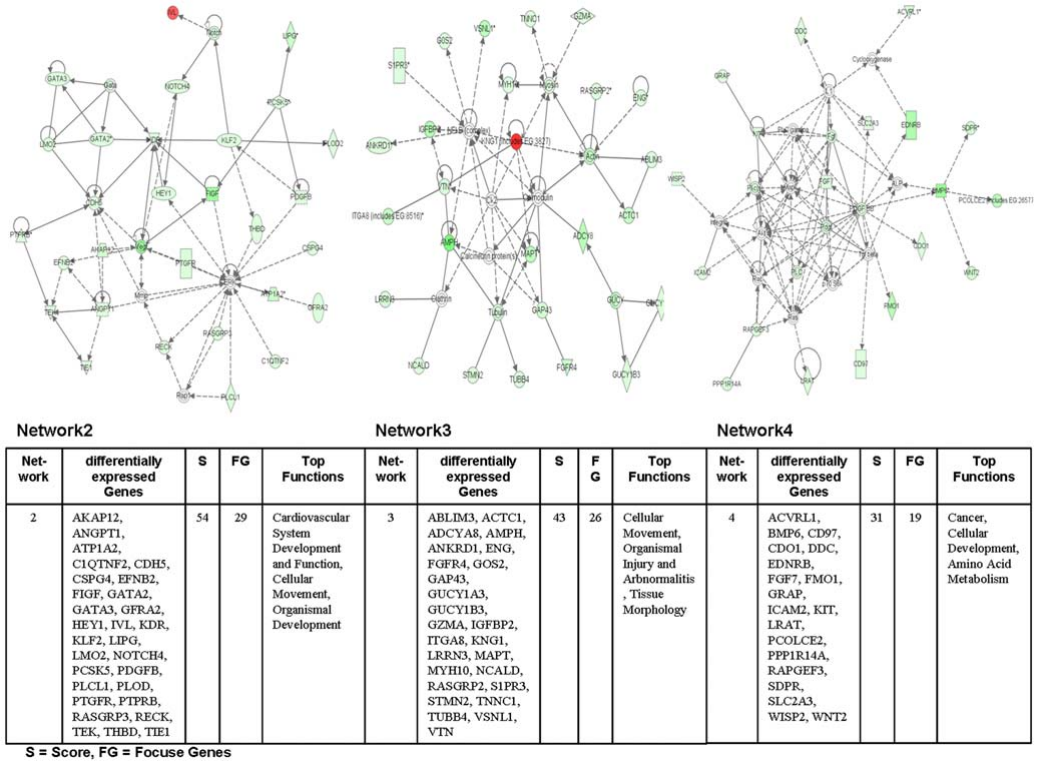
Ingenuity networks exclusively regulated in adenocarcinoma. Ingenuity networks generated by mapping the focus genes that were exclusively regulated in adenocarcinoma.

**Network 2** (Figure 17) consisted of predominantly down regulated genes with the central components Vegf, Erk/Mapk and Pdgf. Notably, Vegf plays pivotal roles in the regulation of angiogenesis and function as a vascular permeability factor to increase microvascular permeability. Importantly, in situ hybridization studies have demonstrated Vegf mRNA to be up-regulated in the majority of human tumors. Only sections of lobular carcinoma of the breast and papillary carcinoma of the bladder failed to show significant Vegf mRNA expression [174]. The biological effects of Vegf are largely mediated by the VEGF receptor tyrosine kinase Kdr (kinase domain region) and c-fos induced growth factor (Figf/Vegfd) all being direct targets of Vegf. Indeed, Vegfd was localized in tumor cells and endothelium in human non-small cell lung carcinoma [175]. The network displays regulation of these molecules as well as Angpt1 (angiopoietin1), which binds and activates the Tek/Tie2 endothelial-specific receptor tyrosine kinase, Efnb2 (ephrinB2) [176], Cdh5 (cadherin 5) and Ptprb (protein tyrosine phosphatase, receptor type, B or vascular endothelial tyrosine phosphatase). In recent works it was reported that activation of Vegf/Vegfr2 and Angiopoietin/Tie2 signalling is modulated by BMP (bone morphogenetic protein) signaling-activation in endothelium [177]. Notably, Bmp6 was strongly down regulated in our transgenic adenocarcinoma model.

Further to pertubations in signalling pathways associated with angiogenesis the network depicts various transcription factors involved in angiogenesis as well.

Specifically, Hey1 (hairy/enhancer-of-split related with YRPW motif 1) inhibits Vegfr2 [151] and indirectly activates p53 through regulation of Mdm2 [152]. Recent work showed that Hey1, through its interplay with Runx2, play an important role in regulating Bmp9 [178]. Moreover, Hey 1 is a primary target of Notch [179] with Notch 4 (Notch gene homolog 4) being down-regulated in our transgenic mouse model. Specifically, Notch proteins are single-pass transmembrane receptors that function as nuclear transcriptional co-activators. Notch signalling is required for vascular development and tumor angiogenesis. In fetal lung development, Notch signalling appears to be essential for the lung to achieve its normal size and Notch 1 and Notch 2 protein are frequently expressed in human non-small cell lung cancer with Notch3 mRNA expression being detected in 7 of 25 NSCLC cell lines [180]. Furthermore, in murine keratinocytes Notch1 signalling stimulates expression of early differentiation markers such as keratin1 and involucrin. IVL (involucrin), which we found up-regulated in adenocarcinoma and encodes a protein of the terminally differentiated keratinocyte. It could be shown that in the absence of mutant p53 involucrin was up-regulated in the human lung adenocarcinoma cell line Anip973 [181].

A further component of the transcriptional network are the GATA binding proteins 2 and 3, Lmo2 (LIM domain only 2) and Klf2 (Kruppel-like factor 2). Note, Gata2 is expressed in the precursor of hematopoietic cells and reduction of Gata2 expression or activity is required for differentiation into hematopoietic cells [182]. Gata3 expression is restricted to T-lymphoid cells and some nonhematopoietic cell types, including embryonic stem cells [183] but Lmo2 is necessary for early stages of hematopoiesis, while the Lmo2 protein plays a central role in angiogenesis [184]. The network implies Klf2 to be targeted by PdgfB (platelet derived growth factor, B polypeptide). Recent studies evidenced PdgfB to regulate Klf2 expression through Src signalling which is also critical for vascular formation [185].

Several metabolic processes are connected in this network, e.g. carbohydrate and lipid metabolism with Pcsk5 catalycing reactions ranging from precursors for growth factors, such as Figf, to cell surface receptors and viral surface glycoproteins [186]. An indirect downstream regulated molecule of Pcsk5 is Lipg (lipase, endothelial) and Plod2 (procollagen lysine, 2-oxoglutarate 5-dioxygenase 2) that catalyzes hydroxylation of lysyl residues in collagens. Recently, Chang et al proposed Plod2 as one of the fibroblast core serum response genes associated with cancer progression [187].

Next to angiogenic signalling key components of the Erk/Mapk1 (mitogen-activated protein kinase 1) are depiceted. Although Erk is not regulated by itself it is a target molecule of, for instance, Figf (c-fos induced growth factor), Thbd (thrombomodulin), Cspg4 (chondroitin sulfate proteoglycan 4), C1QTNF2 (C1q and tumor necrosis factor related protein 2), Plcl1 (phospholipase C-like 1), RasGRP3 (RAS, guanyl releasing protein 3) and Ptgfr (prostaglandin F receptor). A direct target molecule displayed in this network are Reck (reversion-inducing-cysteine-rich protein with kazal motifs) and ATP1A2 (ATPase, Na+/K+ transporting, alpha 2 polypeptide).

A further important member of this network is the prostaglandin F receptor, i.e. a member of the G-protein coupled receptor family that plays central roles in the contraction of smooth muscles of the bronchus and trachea [188]. It was reported that an activation of EGFR-Ras-mitogen-activated protein kinase/Erk kinase (MEK) via the Ptgfr resulted in an increase in Vegf promoter activity, expression of Vegf mRNA, and secretion of VEGF protein [189].

**Network 3** describes interaction partners in the control and the dynamic of the cytoskeleton and the cell mobility in lung tumors. Of all regulated genes in this network only one is up-regulated (Figure 17).

Notably, the up-regulated Kng1 (kininogen 1) generates two isoforms of high molecular weight kininogen (Hmwk) and low molecular weight kininogen (lmwk). Hmwk is important for the assembly of plasma kallikrein. Kallikrein is assumed to play a role in angiogenesis of prostate and breast cancer [190].

Direct down stream target molecules by Kng1 are myosin and F-actin, the latter being a filamentous polymer of the cytoskeleton. In non muscle cells it serves as trail for myosin transporters and is necessary for the cell motility. Motility is important for invasion and metastasis in cancer. F-actin has four partners and besides myosin in this network, their are Ablim3, Actc1 Rasgrp2 and Eng depicted. Indeed, Ablim3 (actin binding LIM protein family, member 3) is an actin binding Zn-finger protein. While Lim domain proteins are known to be regulated in embryonic development and in cancer it is postulated that Ablim3 functions as linkage between the actin cytoskeleton and signaling pathways [191]. Furthermore, Actc1 (actin, alpha, cardiac muscle 1) belongs to the actin sub family of alpha actins which can be found in muscular tissue. Likewise, Rasgrp2 (RAS guanyl releasing protein 2) is a nucleotide (GDP/GTP) exchange factor that activates ras subfamily GTPases. The localization of Rasgrp2 to the membrane seems to be directly associated with the polymerization of F-actin [192]. Furthermore, Eng (endoglin) encodes for a homodimeric transmembrane protein that is part of the Tgf-β receptor complex with high affinity to Tgf-β1 and Tgf-β3. It could be shown that Eng is a possible prognostic marker of prostate cancer [193].

Specifically, myosin, a direct interacting partner with F-actin, is a motor protein. Three regulated genes surround myosin in the network: Myh1 (myosin, heavy polypeptide 10, non-muscle), Tnnc1 (troponin C, cardiac/slow skeletal) and Gzma (granzyme A).

In the same network several partners around NF-kappa-B are displayed, i.e. Vsnl1 (visinin-like 1), G0s2 (G0/G1switch 2), S1pr3 (sphingosine-1-phosphate receptor 3), Ankrd1 (ankyrin repeat domain 1), Igfbp2 (insulin-like growth factor binding protein 2) and Amph (amphiphysin). Notably, Vsnl1 (visinin-like 1) belongs to the visinin/recoverin subfamily of neuronal calcium sensor proteins and is normally expressed in granule cells of the cerebellum. It is responsible for intracellular modulation of signaling pathways. The participation of Vsnl1 in the regulation of proliferative and invasive properties within neuroblastoma has already been suggested [194]. Also, G0s2 (G0/G1switch 2) is suggested to be phosphorylated by protein kinase C and casein kinase II. It is postulated to be a target gene in all-trans-retinoic acid (RA) treatment of acute promyelocytic leukemia [195], where as sphingosine-1-phosphate receptor 3 belongs to the EDG (endothelial differentiation gene) receptor family of g protein coupled receptors. The receptor appears to be the target for sphingosine-1-phosphate in angiogenesis. A further member in this network is ankyrin repeat domain 1 (cardiac muscle), a transcription factor induced by IL-1 and upon TNF-alpha stimulation. In ovarian cancer a connection between expressions level of Ankrd1 and treatment with platinum based chemotherapy has been reported [196], as has been for insulin-like growth factor binding protein 2. Indeed, overexpression of Igfbp2 in transgenic mice led to decreased appearance of dysplastic aberrant crypt foci and inhibited the growth of adenomas [197].

Furthermore, amphiphysin that encodes a protein involved in clathrin-mediated endocytosis is displayed. It may function as linker of clathrin coat proteins and membrane curvature sensor. Regulation of endocytosis may impact Cdk5 dependent phosphorilation of amphiphysin [198]. Amph is further connected to tubulin and Vtn (vitronectin) which is connected to the previously described Kng1 and Itga8 (integrin alpha 8). Noteworthy, leucine rich repeat protein 3 facilitates the Egf induced MAPK phosphorylation and may be engaged in internalization of EGF by clathrin-coated vesicles while neurocalcin delta being a part of the neuronal calcium sensor family of calcium-binding proteins. It is postulated to have a regulatory effect on G-protein coupled receptors.

Additionally, the down-regulated tubulin gene is positioned in the center of three likewise regulated genes: Stmn2 (stathmin-like 2), Tubb4 (tubulin, beta 4) and Mapt (microtubule-associated protein tau). Tubulin forms the microtubuli which is a part of the cytoskeleton and mainly consists of two forms alpha and beta tubulin. The microtubule is important for mitotic spindle formation and therefore targeted in cancer therapy. Stathmin-like 2 is a microtubule regulator that seems to destabilize the microtubuli. Tubb4 (tubulin, beta 4) is an isoform of beta tubulin and may be necessary for mitosis [199]. Finally, in this network downstream partners of calmodulin are connected, e.g. Gucy1a3 (guanylate cyclase 1, soluble, alpha 3), Gucy1b3 (guanylate cyclase 1, soluble, beta 3) and Adcy8 (adenylate cyclase 8). Notably, while Gucy catalyzes the conversion of GTP to cGMP and acts as second messenger adenylate cyclase catalyses the formation of cAMP out of ATP and is part of G-protein dependent signaling pathways.

The **fourth** network in adenocarcinomas (Figure 17) depicts several ligands of tyrosine kinases like Fgf7 (fibroblast growth factor 7) and Pdgfbb (platelet derived growth factor, B polypeptide) as regulated. Both growth factors are involved in angiogenesis. Specifically, fibroblast growth factor-7, also known as keratinocyte growth factor, is involved in the regulation of proliferation and differentiation of alveolar epithelial type II (AEII) cells [200] and bronchiolar epithelial cells [201]. Moreover, it was shown that Fgf7 stimulate the synthesis of surfactant components in rat fetal ATII cells, thereby promoting maturation of the lung epithelium [202]. Additionally, overexpression or loss of expression of Fgf7 and of its specific receptor was reported for a variety of tumours including lung cancer [203]. Further target molecules of PdgfB in this network are EdnrB (endothelin receptor type B), Fom1 (flavin-containing monooxygenase1) and Bmp6 (bone morphogenetic protein 6) all of which were strongly down regulated. Ednrb is a G protein-coupled receptor which activates a phosphatidylinoitol-calcium second messenger system and mediates biological responses from a variety of stimuli, including growth factors, such as PdgfB, vasoactive polypeptides, neurotransmitters, hormones, and phospholipids while EdnrB receptor activation leads to bronchoconstriction. Importantly, downregulation of endothelin receptor type B was observed in human tumor cells [204]. A further molecule of this network is flavin-containing monooxygenase 1 that catalyzes oxidation of many molecules. It was shown that in vascular smooth muscle cells from rat aorta PdgfB increased the expression of rat Fmo1 mRNA [205]. Likewise, bone morphogenetic protein 6 is a member of the TGF-β superfamily and crucial for all stages of embryonic development, including regulation of lung development and airway branching. This molecule mediates antigrowth signalling by suppressing proliferation. Recent evidence suggests epigenetic inactivation of Bmp6 in NSCLC [206]. Increasing evidence is also suggestive for crosstalk between BMP antigrowth signalling and Ras/MAPK progrowth signalling. In the present study, we did not observe a connection between BMP and MAPK at the transcriptional level [207]. Nonetheless, there appears to be cross-talk between the BMP and Wnt pathway. Thus, Wnt2 (wingless-related MMTV integration site 2) is depicted as a downstream molecule of Bmp6. So far such cross-talk has only been observed in cultured endothelial cells where Bmp6 protein increases the expression of Wnt2 mRNA [208]. Members of the Wnt pathway were reported to be regulated in a number of cancers as well as embryogenesis. Notably, Wnt2 is overexpressed in human colorectal carcinomas but only up-regulated in a small subset of prostate cancer [209], but is overexpressed in primary NSCLC and plays a role as mediator of apoptosis in several cancers of epithelial origin [210]. A further downstream target of Bmp6 is Sdpr (serum deprivation response). This protein is a substrate of protein kinase C and binds phosphatidylserine (PS) in a calcium-independent manner. Recently, it was shown that Sdpr expression is suppressed in tumors of breast, kidney and prostate [211], but until now regulation of this gene was not associated with lung cancers.

A direct target of PdgfB is phospholipase C (Plc). PLC isozymes catalyze the hydrolysis of phosphatidylinositol 4,5-bisphosphate [PtdIns(4,5)P2] to inositol 1,4,5-trisphosphate and diacylglycerol. These two second messengers activate the serine/threonine-specific protein kinase C (Pkc), which was repressed in adenocarcinomas and functions as modulater for the release of calcium from intracellular stocks. Altered expression of several Plc family members was reported for human cancers. Specifically, Plc is highly expressed in breast carcinomas, and in metastatic colorectal tumor cell lines [212]. However, a greatly reduced mRNA expression of the Plc-L gene was observed in a large portion of lung carcinoma cells [213], [214]. It was shown that PLC could be activated by members of the Ras and Rho families of guanosine trisphosphatases (GTPases) such as RapGEF3 (Rap guanine nucleotide exchange factor3), which we observed to be regulated as well. Downstream of Plc is the serine/threonine-specific protein kinase C (Pkc). Deregulation of certain types of PKCs plays a role in human lung carcinogenesis through loss of control of cell growth and/or differentiation. Recently, atypical PKCzeta was shown to be involved in the chemotaxis and cell adhesion of NSCLC cells with integrin β1 facilitating this process [215]. Moreover, overexpression of the Pkc gene in colon carcinoma cells causes growth inhibition and decreased tumorigenicity [216]. CD97, a glycoprotein that is present on the surface of most activated leukocytes and spans the membrane seven times is a further member of this network. To date, three ligands have been identified for CD97. One ligand for CD97 is the α5β1 integrin. It was proposed that CD97 plays a role in migration and adhesion of tumor cells [217].

Further regulated genes in network 4 are Icam2, Wisp2, Grap and Kit. ICAM2 (intracellular adhesion molecule 2) a member of the Ig superfamily of cell surface proteins that may act as an activator of the PI3K/AKT pathway to promote cell migration [218] eventhough expression levels of ICAM2 in peritoneal metastases tend to be decreased as compared to primary lesions. Thus, it was hypothesized that ICAM2 enhances the adhesion and activation of immune cells such as natural killer cells, resulting in a reduction of metastasis [219]. Additionally, WNT1 inducible signaling pathway protein 2 was identified as growth factors. WISP proteins bind to glycosaminoglycans and may function in angiogenesis, stem cell differentiation, and cancers. It was shown that WISP2 mRNA and protein levels were significantly reduced as breast cancer progresses from a noninvasive to invasive type and were almost undetectable in poorly differentiated cancers [220]. Likewise, GRB2-related adaptor protein (Grap), plays a central role in signalling patways due to interaction with other signalling molecules such as p36/38, Slp76 (lymphocyte cytosolic protein 2), Shc (src homology 2 domain-containing transforming protein C1) and Sos (son of sevenless homolog 1). Importantly, Grap appears to be a negative regulator specific for the Erk pathway [221] but little is known about its role in cancer. Additionally, we found the kit oncogene in this network. Binding of stem cell factor (SCF) to Kit receptor tyrosine kinase activates multiple signal transduction components, leading to the activation of MAPK pathway [222], phosphatidylinositol 3-kinase, and Jak/Stat signalling [223]. The tyrosine kinase receptor c-kit and its ligand stem cell factor are co-expressed in various solid tumors and were identified in 64% of primary adenocarcinomas of the lung [224]. However, expression of c-Kit progressively decreases during local tumor growth and invasion of human melanomas [225]. In this regard one group reported that c-kit expression was decreased in advanced stages of ovarian cancer and was associated with decreased survival [226].

Further members of this network are Ddc (dopa decarboxylase), Acvrl1 (activin A receptor, type II-like 1) and Lrat (lecithin-retinol acyltransferase), but are not directly linked to other regulated molecules in this network.

Taken collectively, we constructed several networks that were associated either specifically with dysplasia (Supplementary Figure S5) or adenocarcinomas. In dysplasia, we observed strongly up-regulation of Hnf4α and some of its target molecules. Especially target genes of Hnf4α are regulated which encoded proteins involved in metabolic processes. Second, we found several transcription factors overexpressed in dysplasia. In strong contrast, down regulation of many genes involved in angiogenesis, early and restructuring of the cytoskeleton was a prominent feature of adenocarcinomas.

Taken collectively, our study enabled an identification of a limited set of molecules particularly associated with either dysplasia or advanced MAPK induced adenocarcinomas. The differential gene expression profiles of dysplasia and adenocarcinoma enebled an identification of specific molecules, to allow for better molecular classification of dysplasia and an improved understanding of the biology in the progression of disease. Improvement in the survival rate of patients with adenocarcinoma will depend upon better diagnosis. The aplication of cancer genomics thus allowed for the construction of gene regulatory networks for a better understanding of tumor biology and the validation of a wealth of novel targets to be explored in innovative treatment regimes of lung cancer. The role of non-coding RNA in lung adenocarcinomas will be the subject of future studies [227].

## Materials and Methods

### SP-C/c-raf model

SP-C/c-raf transgenic mice were obtained from the laboratory of Prof. Ulf Rapp (University of Würzburg, Germany), who bred the mice in the C57BL/6/DBA/2 hybrid background. We kept the SP-C/c-raf transgenic mice in the C57BL/6 background for at least five generations.

Lung cancer samples were derived from 5 SP-C/c-raf mice (aged 12–14 months); unaltered lung tissue were always isolated from 5 transgenic mice (aged 5–7 months). Endogenous normal lung tissue was studied of 5 non-transgenic mice (aged 7–10 months). The non-transgenic littermates (wild-type) served as control for transgenic effects.

Mice were sacrificed and the lung tissues were immediately frozen on dry ice and stored at −80°C until further analysis.

The histopathological diagnosis was based on routinely processed hematoxylin-eosin stains.

### Microdissection (LMPC – Laser Microbeam Microdissection and Laser Pressure Catapulting)

From each frozen lung tissue 10-µm thick sections were prepared and transferred on polyethylene napthalate foil-covered slides (Zeiss, P.A.L.M. Microlaser Technologies GmbH, Bernried, Germany).

The sections were fixed in methanol/acetic acid and stained in hematoxylin. The desired cells were microdissected using the PALM MicroLaser systems (Zeiss, P.A.L.M. Microlaser Technologies GmbH, Bernried, Germany) and collected in an adhesive cap (Zeiss, P.A.L.M. Microlaser Technologies GmbH, Bernried, Germany). Microdissected cells were resuspended in a guanidine isothiocyanate-containing buffer (RLT buffer from RNeasy MikroKit, Qiagen, Santa Clarita, CA, USA) with 10 µl/ml β-mercaptoethanol to ensure isolation of intact RNA. Approximately an area of 6×10^6^ µm^2^ were pooled from a specific layer of interest in the same animal and used for RNA extraction.

Following microdissection, total RNA-extraction was performed with the RNeasy Micro Kit (RNeasy MicroKit Qiagen, Santa Clarita, CA, USA) according to the manufacturer's instruction. A standard quality control of the total RNA was performed using the Agilent 2100 Bioanalyzer (Agilent Technologies, Palo Alto, USA).

### cRNA labeling and hybridization to microarrays

Total RNA (median: 175 ng; range: 150–200 ng) was used to generate biotin-labeled cRNA (10 µg) by means of Message Amp aRNA Premium Amplification Kit (Ambion, Austin, TX). Quality control of cRNA was performed using a bioanalyzer (Agilent 2001 Biosizing, Agilent Technologies). Following fragmentation, labeled cRNA of each sample was hybridized to Affymetrix GeneChip® Mouse Genome 430 2.0 Arrays covering over 34.000 genes and stained according to the manufacturer's instructions.

### Quantification, normalization and statistical analysis

Array data was normalized using scaling or per-chip normalization to adjust the total or average intensity of each array to be approximately the same.

Microarray chips were analyzed by the GCOS (GeneChip Operating Software) from Affymetrix with the default settings except that the target signal was set to 500 and used to generate a microarray quality control and data report.

CEL files exported from GCOS were uploaded into ArrayTrack software (National Center for Toxicological Research, U.S. FDA, Jefferson, AR, USA (NCTR/FDA)) and normalized using Total Intensity Normalization after subtracting backgrounds for data management and analysis. ArrayTrack software includes some tools common to other bioinformatics software (e.g., ANOVA, T-test and SAM).

### SAM

To compare the normalized data from dysplasia, normal lung tissue from transgenic mouse, tumor cells and non-transgenic of different mice, we used the Significance Analysis of Microarrays (SAM) algorithm (ArrayTrack), which contains a sliding scale for false discovery rate (FDR) of significantly up- and down-regulated genes [228]. All data were permuted 100 cycles by using the two classes, unpaired data mode of the algorithm. As cut-off for significance an estimated FDR of 0.001 was chosen. Moreover, a cut-off for fold-change of differential expression of 2 was used. The full description of the Extraction protocol, labeling and hybridization protocol and data processing is obtainable in the GEO DATA base under http://www.ncbi.nlm.nih.gov/geo/ (accession number GSE14277).

### Principal component analysis (PCA) and hierarchical gene cluster analysis (HCA)

Two clustering approaches were used to determine components of variation in the data in this study as follows.

A) Principal-component analysis (PCA) that was used to obtain a simplified visualization of entire datasets. PCA is a useful linear approach to obtain a simplified visualization of entire datasets, without losing experimental information (variance). PCA allowed the dimension of complex data to be reduced and highlights the most relevant features of a given dataset to be highlighted.

B) Hierarchical gene clustering (HCA) where the data points were organized in a phylogenetic tree in which the branch lengths represent the degree of similarity between the values. After normalisation and SAM analysis a total of 3246 significant genes were used for hierarchical clustering.

### Functional analysis of the significant genes with IPA

Lists of significantly differentially expressed genes were uploaded to Ingenuity Pathways Analysis (IPA, Ingenuity Systems Inc., Redwood City, CA, USA) (www.Ingenuity.com) and functional annotation and pathway analysis was performed. IPA is a commercial, web-based interface that uses a variety of computational algorithms to identify and establish cellular networks that statistically fit the input gene list and expression values from experiments. The analysis uses a database of gene interactions culled from literature and updated every quarter of the year.

Additionally, Venn diagrams were used to examine the overlap of resulting lists of genes differentially expressed between the different sample sets.

### Quantitative real-time PCR

Corroboration of RNA expression data was performed by real-time PCR using the ABI PRISM 7500 Sequence Detection System Instrument (Applied Biosystems, Applera Deutschland GmbH, Darmstadt, Germany). Total RNA (200 ng) underwent reverse transcription using an Omniscript RT Kit (Qiagen, Santa Clarita, CA, USA) according to the manufacturer's instruction. PCR reactions were performed according to the instructions of the manufacturer using commercially available assays-on-demand (Applied Biosystems, Applera Deutschland GmbH, Darmstadt, Germany). CT values were calculated by the ABI PRISM software and relative gene expression levels were expressed as the difference in CT values of the target gene and the control gene Actin beta.

### Immunohistochemistry

Each tumor section (8 µm in thickness) was deparaffinized in roti-histol for 2 times 8 minutes, these were dehydrogenated by means of a descending alcohol row. The following incubation steps were accomplished: 2 times 3 minutes in 96% ethanol, 2 times 2 minutes in 70% Ethanol, and 2 minutes in Aqua dest. The pre-treated slices were heated in a autoclave for 15 min in citrate buffer submitted of an antigen retrieval before the colouring first. For blocking endogenous peroxidase activity the slices covered for 30 minutes with 3% hydrogen peroxide/Methanol peroxidase blocking solution. After a wash step, the slices were incubated with the primary polyclonal anti-body against Cldn2 (Biomol GmbH, Germany), Mcc (Proteintech Group, Inc., IL, USA), S100a14 (Applied Biological Materials Inc. Richmond, BC Canada), Cdh5, Fetub, Fst, Orm1, RasGRF1, Hnf4α and Reck (Santa Cruz, Santa Cruz Biotechnologys Inc., CA, USA) for 45 minutes. After washing, a streptavidin horseradish peroxidase detection kit (Envision DAKO, Hamburg, Germany) containing 3,3′-diaminobenzidine solution as substrate was used for immunohistochemical staining according to the manufacturer's instructions. Harris Hämatoxylin was used as the counterstaining. The specificity of the immunostaining was confirmed by positive and negative control staining. For negative controls we used mouse nonimmune immunoglobulin G or blocking peptide instead of the primary antibody. For the positive control staining we use normal tissue with known positive immunoreactions to the antibodies (Cldn2, Fst, Mcc, S100a14 = colon; Fetub, Orm1, Reck, RasGRF1, Hnf4α = liver and Cdh5 = heart).

## Supporting Information

Figure S1 Ingenuity - Canonical Pathways: adenocarcinoma vs transgenic. This figure shows the canonical pathways which were overrepresented in the group of significantly regulated genes in adenocarcinoma versus transgenic mice.
(0.71 MB TIF)

Figure S2 Ingenuity - Canonical Pathways: adenocarcinoma vs non-transgenic. This figure shows the canonical pathways which were overrepresented in the group of significantly regulated genes in adenocarcinoma versus non-transgenic mice.
(2.11 MB TIF)

Figure S3 Corroboration by quantitative real-time PCR (Part I). Real-time PCR curves of seven genes assessed by Taqman technology as well as of the reference gene ACTB of a representative experiment are shown. The differences of the Ct values of target and ACTB (deltaCT) are indicated. The smaller the deltaCT, the higher the relative expression level of the target mRNA.
(6.74 MB TIF)

Figure S4 Corroboration by quantitative real-time PCR (Part II). Real-time PCR curves of seven genes assessed by Taqman technology as well as of the reference gene ACTB of a representative experiment are shown. The differences of the Ct values of target and ACTB (deltaCT) are indicated. The smaller the deltaCT, the higher the relative expression level of the target mRNA.
(6.76 MB TIF)

Figure S5 Gene networks exclusively regulated in dysplasia. Ingenuity networks generated by mapping the focus genes that were associated in dysplasia (descriptions see Fig. 6).
(3.62 MB TIF)

Table S1 List of genes with changed expressions that are significantly overexpressed in adenocarcinoma versus non-altered transgenic mice: 473 significantly regulated genes. This table shows the RefSeq transcript IDs, Unigene IDs, gene titles, gene symbols, and fold changes of the significantly regulated genes.
(0.65 MB DOC)

Table S2 List of genes with changed expressions that are significantly over- or under-expressed in adenocarcionam versus non-transgenic mice: 541 significantly regulated genes. This table shows the RefSeq transcript IDs, Unigene IDs, gene titles, gene symbols, and fold changes of the significantly regulated genes.
(0.74 MB DOC)

Table S3 List of genes with changed expressions that are significantly over- or under-expressed in unaltered transgenic versus non-transgenic mice: 18 significantly regulated genes. This table shows the RefSeq transcript IDs, Unigene IDs, gene titles, gene symbols, and fold changes of the significantly regulated genes.
(0.05 MB DOC)

Text S1 Description of gene regulated networks associated in dysplasia.
(0.04 MB DOC)
