# Supplementary figures and images for: Cancer Genomics Identifies Regulatory Gene Networks Associated with the Transition from Dysplasia to Advanced Lung Adenocarcinomas Induced by c-Raf-1
Source: PLoS One. 2009 Oct 8;4(10):e7315. doi: 10.1371/journal.pone.0007315 (PMC2754338; doi:10.1371/journal.pone.0007315)

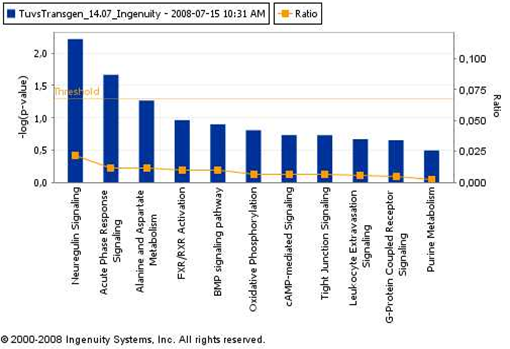

Supplement: Figure S1 — Ingenuity - Canonical Pathways: adenocarcinoma vs transgenic. This figure shows the canonical pathways which were overrepresented in the group of significantly regulated genes in adenocarcinoma versus transgenic mice. (0.71 MB TIF) [file pone.0007315.s001.tif]

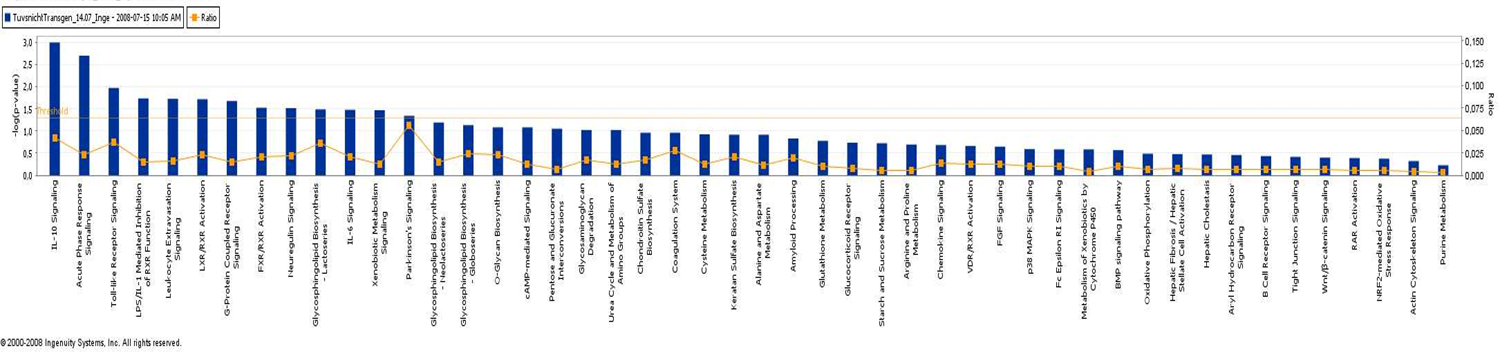

Supplement: Figure S2 — Ingenuity - Canonical Pathways: adenocarcinoma vs non-transgenic. This figure shows the canonical pathways which were overrepresented in the group of significantly regulated genes in adenocarcinoma versus non-transgenic mice. (2.11 MB TIF) [file pone.0007315.s002.tif]

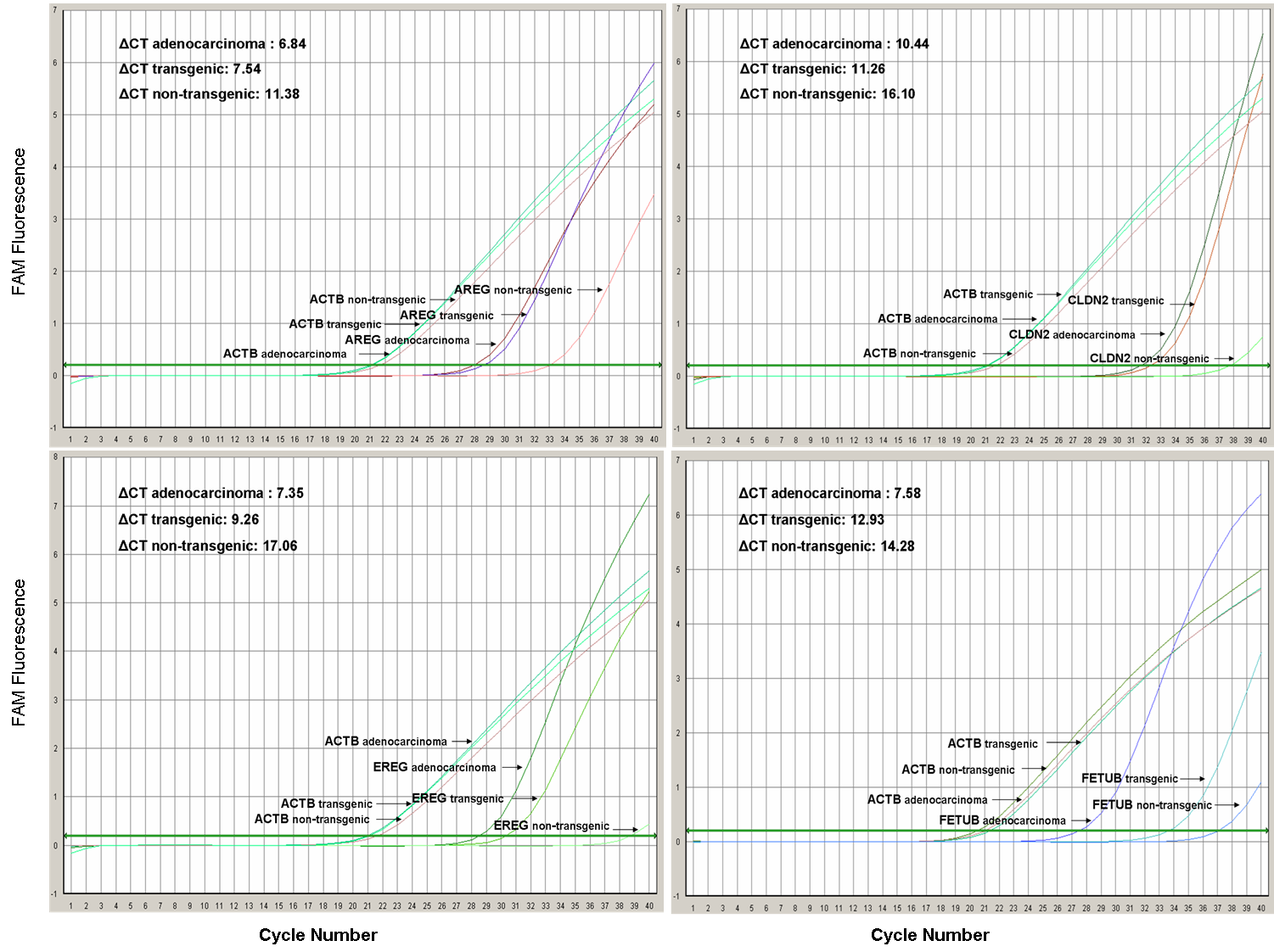

Supplement: Figure S3 — Corroboration by quantitative real-time PCR (Part I). Real-time PCR curves of seven genes assessed by Taqman technology as well as of the reference gene ACTB of a representative experiment are shown. The differences of the Ct values of target and ACTB (deltaCT) are indicated. The smaller the deltaCT, the higher the relative expression level of the target mRNA. (6.74 MB TIF) [file pone.0007315.s003.tif]

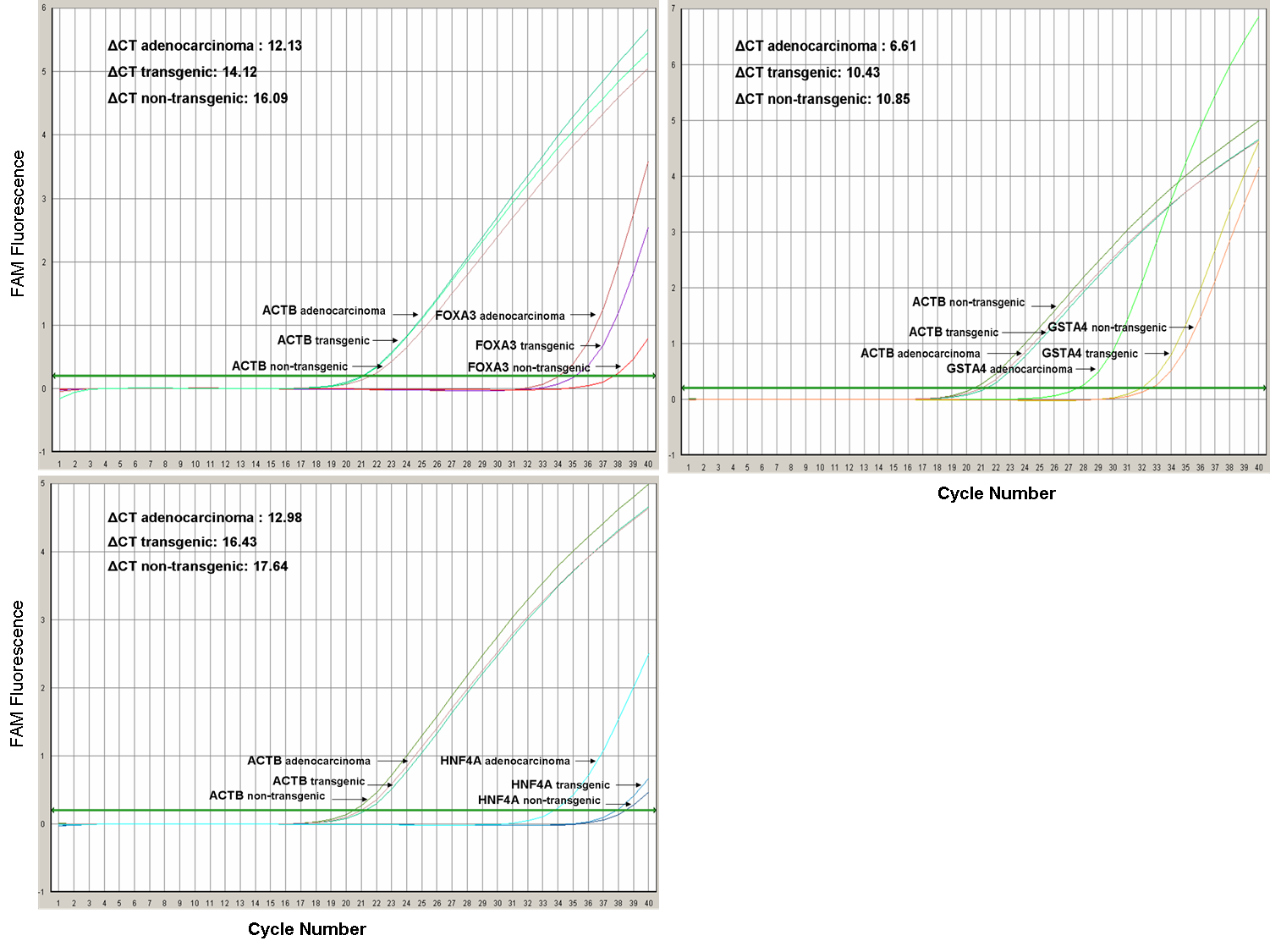

Supplement: Figure S4 — Corroboration by quantitative real-time PCR (Part II). Real-time PCR curves of seven genes assessed by Taqman technology as well as of the reference gene ACTB of a representative experiment are shown. The differences of the Ct values of target and ACTB (deltaCT) are indicated. The smaller the deltaCT, the higher the relative expression level of the target mRNA. (6.76 MB TIF) [file pone.0007315.s004.tif]

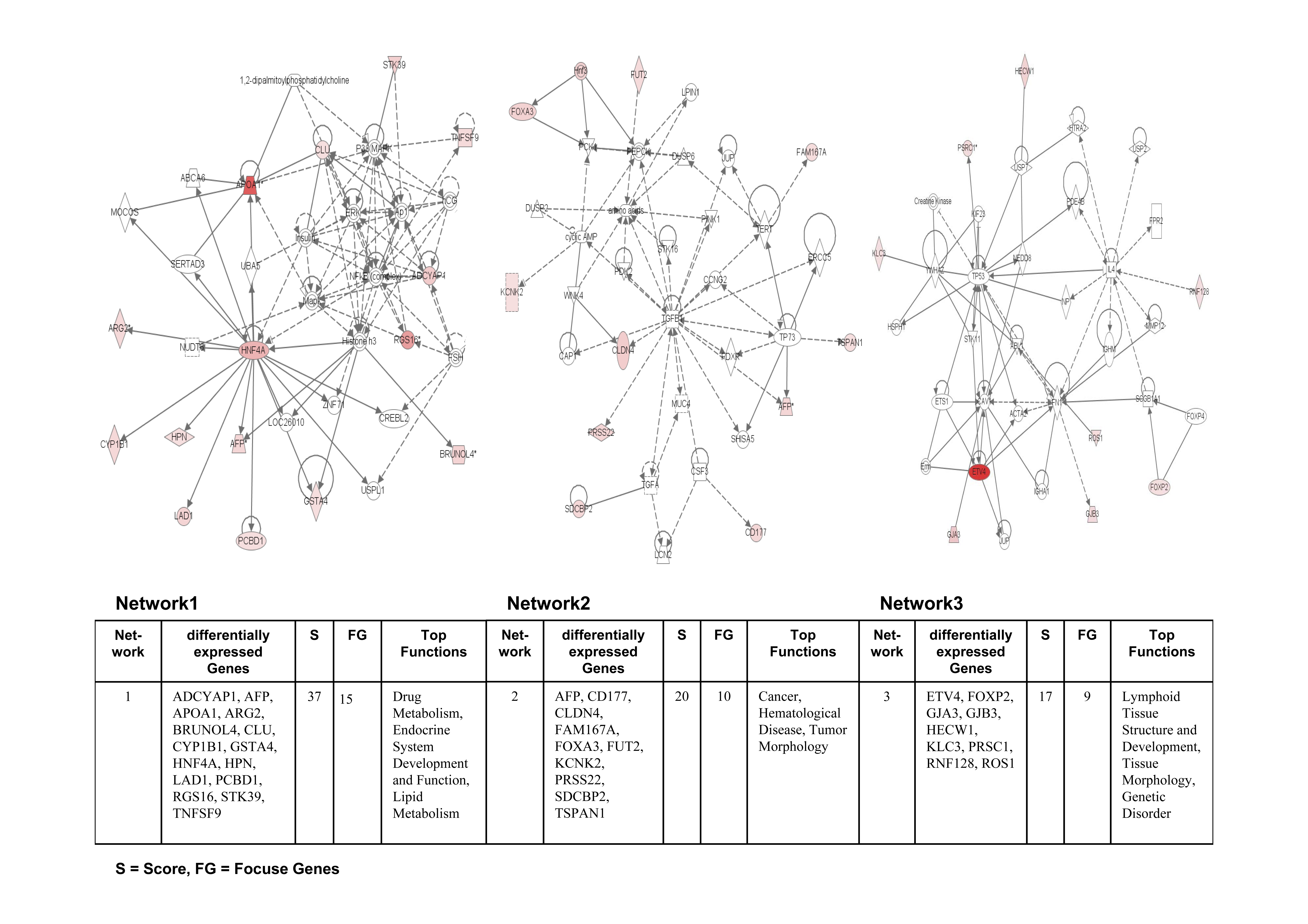

Supplement: Figure S5 — Gene networks exclusively regulated in dysplasia. Ingenuity networks generated by mapping the focus genes that were associated in dysplasia (descriptions see Fig. 6). (3.62 MB TIF) [file pone.0007315.s005.tif]
