# Supplementary material for: Cancer Genomics Identifies Regulatory Gene Networks Associated with the Transition from Dysplasia to Advanced Lung Adenocarcinomas Induced by c-Raf-1
Source: PLoS One. 2009 Oct 8;4(10):e7315. doi: 10.1371/journal.pone.0007315 (PMC2754338; doi:10.1371/journal.pone.0007315)
Supplement: Table S1 — List of genes with changed expressions that are significantly overexpressed in adenocarcinoma versus non-altered transgenic mice: 473 significantly regulated genes. This table shows the RefSeq transcript IDs, Unigene IDs, gene titles, gene symbols, and fold changes of the significantly regulated genes. (0.65 MB DOC) [file pone.0007315.s006.doc]

| **Genename** | **Gene Title** | **Fold Change** | **Gen_id_mfr** | **RefSeq Transcript ID** |
| --- | --- | --- | --- | --- |
| Cbln1 | cerebellin 1 precursor protein | 33,20 | 1423287_at | NM_019626 |
| 1810036H07Rik | RIKEN cDNA 1810036H07 gene | 30,28 | 1453132_a_at | NM_197999 |
| Ndg1 /// LOC623189 | KH domain containing 1A | 29,43 | 1455423_at | NM_010882 |
| Orm1 | orosomucoid 1 | 28,14 | 1451054_at | --- |
| Pthlh | parathyroid hormone-like peptide | 26,69 | 1422324_a_at | NM_029928 |
| Cbln1 | cerebellin 1 precursor protein | 26,02 | 1423286_at | NM_019626 |
| Kng1 | kininogen 1 | 25,79 | 1416676_at | NM_010698 |
| Pkhd1 | polycystic kidney and hepatic disease 1 | 24,17 | 1419820_at | NM_019588 |
| Chl1 | Cell adhesion molecule with homology to L1CAM | 20,46 | 1435190_at | NM_031258 |
| Atp6v0a4 | ATPase, H+ transporting, lysosomal V0 subunit A isoform 4 | 19,06 | 1422030_at | --- |
| Areg | amphiregulin | 18,04 | 1421134_at | NM_175535 |
| Ereg | epiregulin | 15,29 | 1419431_at | XM_283813 |
| Fetub | fetuin beta | 15,19 | 1449555_a_at | NM_008008 |
| Ivl | involucrin | 14,70 | 1439878_at | NM_010612 |
| Ccdc83 | coiled-coil domain containing 83 | 14,03 | 1453425_at | NM_173556 |
| Itih2 | inter-alpha trypsin inhibitor, heavy chain 2 | 13,81 | 1417618_at | NM_008412 |
| 9130213B05Rik | RIKEN cDNA 9130213B05 gene | 13,45 | 1424214_at | NM_145562 |
| Cldn2 | claudin 2 | 13,44 | 1417231_at | NM_013805 |
| D630002J15Rik | RIKEN cDNA D630002J15 gene | 12,67 | 1453480_at | NM_007826 |
| Ankrd22 | ankyrin repeat domain 22 | 12,12 | 1453239_a_at | --- |
| Gpc6 | Glypican 6 | 11,47 | 1428774_at | NM_153581 |
| Rasgrf1 | RAS protein-specific guanine nucleotide-releasing factor 1 | 11,36 | 1435614_s_at | NM_011245 |
| Ptprn /// LOC669060 | Protein tyrosine phosphatase, receptor type, D | 11,04 | 1416588_at | NM_011215 |
| Pcsk6 | proprotein convertase subtilisin/kexin type 6 | 11,03 | 1426981_at | NM_008804 |
| 9130213B05Rik | RIKEN cDNA 9130213B05 gene | 11,01 | 1428891_at | XM_140198 |
| 1110006E14Rik | RIKEN cDNA 1110006E14 gene | 10,98 | 1431094_at | XM_358515 |
| Gtl2 | GTL2, imprinted maternally expressed untranslated mRNA | 10,07 | 1452183_a_at | NM_144513 |
| Ankrd37 | ankyrin repeat domain 37 | 9,98 | 1436538_at | NM_001039562 |
| Cbln1 | cerebellin 1 precursor protein | 9,92 | 1423288_s_at | --- |
| Gtl2 /// Lphn1 | GTL2, imprinted maternally expressed untranslated mRNA /// latrophilin 1 | 9,30 | 1452905_at | NM_021896 |
| Tmem54 | transmembrane protein 54 | 8,84 | 1417895_a_at | NM_025452 |
| Ptprn2 | RIKEN cDNA 4930425H11 gene | 8,70 | 1435968_at | --- |
| St8sia6 | ST8 alpha-N-acetyl-neuraminide alpha-2,8-sialyltransferase 6 | 8,26 | 1456440_s_at | NM_145838 |
| Ckmt1 | creatine kinase, mitochondrial 1, ubiquitous | 8,23 | 1417089_a_at | NM_016675 |
| Sprr1a | small proline-rich protein 1A | 8,23 | 1449133_at | NM_009181 |
| Rian | RNA imprinted and accumulated in nucleus | 8,08 | 1452899_at | --- |
| St8sia6 | ST8 alpha-N-acetyl-neuraminide alpha-2,8-sialyltransferase 6 | 7,66 | 1456147_at | --- |
| --- | Transcribed locus | 7,38 | 1441971_at | --- |
| Wdr16 | WD repeat domain 16 | 7,30 | 1429552_at | NM_145054 |
| Rasgrf1 | RAS protein-specific guanine nucleotide-releasing factor 1 | 7,02 | 1422600_at | --- |
| S100a14 | S100 calcium binding protein A14 | 6,85 | 1449166_at | NM_009122 |
| Oact1 | O-acyltransferase (membrane bound) domain containing 1 | 6,43 | 1435323_a_at | NM_008760 |
| Fst | follistatin | 6,37 | 1434458_at | NM_019503 |
| Adssl1 | adenylosuccinate synthetase like 1 | 6,36 | 1449383_at | --- |
| Akr1c19 | aldo-keto reductase family 1, member C19 | 6,03 | 1455454_at | NM_009653 |
| Adora1 | adenosine A1 receptor | 5,99 | 1435495_at | NM_013462 |
| Cdsn | corneodesmosin | 5,97 | 1444607_at | NM_139206 |
| Btbd11 | BTB (POZ) domain containing 11 | 5,11 | 1428377_at | NM_021426 |
| Gm92 | gene model 92, (NCBI) | 0,29 | 1436962_at | NM_008137 |
| --- | --- | 0,28 | 1454734_at | --- |
| Ghr | Growth hormone receptor | 0,28 | 1451501_a_at | NM_153175 |
| Plce1 | phospholipase C, epsilon 1 | 0,27 | 1452398_at | XM_129785 |
| Lmo2 | LIM domain only 2 | 0,27 | 1454086_a_at | --- |
| Lysmd2 | LysM, putative peptidoglycan-binding, domain containing 2 | 0,27 | 1428626_at | NM_174857 |
| Cspg4 | chondroitin sulfate proteoglycan 4 | 0,26 | 1423341_at | NM_023785 |
| Sept4 | septin 4 | 0,26 | 1448729_a_at | --- |
| Phactr1 | phosphatase and actin regulator 1 | 0,26 | 1439022_at | NM_001005740 |
| 1110006O17Rik | RIKEN cDNA 1110006O17 gene | 0,26 | 1437451_at | NM_026271 |
| Klk8 | kallikrein 8 | 0,26 | 1419722_at | NM_023125 |
| Armcx1 | armadillo repeat containing, X-linked 1 | 0,25 | 1425914_a_at | NM_181728 |
| Sema3c | sema domain, immunoglobulin domain (Ig), short basic domain, secreted, (semaphorin) 3C | 0,25 | 1429348_at | NM_011348 |
| Tm4sf1 | transmembrane 4 superfamily member 1 | 0,25 | 1450958_at | NM_008536 |
| Pdgfb | platelet derived growth factor, B polypeptide | 0,25 | 1450414_at | NM_016798 |
| Itga1 | integrin alpha 1 | 0,25 | 1455251_at | NM_001001309 |
| Plxdc1 | plexin domain containing 1 | 0,25 | 1424902_at | NM_024286 |
| Des | desmin | 0,24 | 1426731_at | NM_138650 |
| Impdh1 | inosine 5'-phosphate dehydrogenase 1 | 0,24 | 1423239_at | --- |
| Adcy4 | adenylate cyclase 4 | 0,24 | 1418098_at | NM_009623 |
| 6330417G04Rik | RIKEN cDNA 6330417G04 gene | 0,24 | 1440849_at | XM_484234 |
| Slc16a9 | solute carrier family 16 (monocarboxylic acid transporters), member 9 | 0,24 | 1429727_at | NM_053195 |
| Ms4a4b | membrane-spanning 4-domains, subfamily A, member 4B | 0,24 | 1423467_at | NM_018857 |
| Zfp697 | zinc finger protein 697 | 0,24 | 1440999_at | NM_172863 |
| Prkce | protein kinase C, epsilon | 0,23 | 1452878_at | NM_011104 |
| Prkce | Protein kinase C, epsilon | 0,23 | 1437861_s_at | NM_008926 |
| Eml1 /// LOC634102 | LOC634102 similar to echinoderm microtubule associated protein like 1 | 0,23 | 1428321_at | NM_139138 |
| Efnb2 | ephrin B2 | 0,23 | 1419639_at | NM_178444 |
| Sox13 | SRY-box containing gene 13 | 0,23 | 1450191_a_at | NM_011446 |
| Ntn1 | netrin 1 | 0,23 | 1454974_at | NM_153546 |
| 3732412D22Rik | RIKEN cDNA 3732412D22 gene | 0,23 | 1435321_at | NM_001001980 |
| --- | --- | 0,23 | 1444088_at | --- |
| Eng | endoglin | 0,23 | 1417271_a_at | NM_007932 |
| Aldh1a1 | aldehyde dehydrogenase family 1, subfamily A1 | 0,23 | 1416468_at | NM_175007 |
| Ndn | necdin | 0,23 | 1415923_at | NM_010882 |
| 9630050M13Rik | RIKEN cDNA 9630050M13 gene | 0,22 | 1438402_at | --- |
| Ncald | neurocalcin delta | 0,22 | 1417569_at | NM_134094 |
| Palld | palladin, cytoskeletal associated protein | 0,22 | 1427228_at | NM_011864 |
| Ppap2b | phosphatidic acid phosphatase type 2B | 0,22 | 1448908_at | NM_026731 |
| Hsd11b1 | hydroxysteroid 11-beta dehydrogenase 1 | 0,22 | 1449038_at | NM_028306 |
| Ddah1 | Dimethylarginine dimethylaminohydrolase 1 | 0,22 | 1454995_at | NM_026993 |
| Tnnc1 | troponin C, cardiac/slow skeletal | 0,21 | 1418370_at | NM_172913 |
| Dmn | desmuslin | 0,21 | 1455610_at | NM_013811 |
| Nsg1 | neuron specific gene family member 1 | 0,21 | 1423055_at | --- |
| Ankrd47 | KN motif and ankyrin repeat domains 3 | 0,21 | 1456629_at | NM_009704 |
| Gp9 | glycoprotein 9 (platelet) | 0,21 | 1419720_at | NM_016697 |
| Plekhk1 | pleckstrin homology domain containing, family K member 1 | 0,21 | 1418108_at | NM_011961 |
| Ndn | necdin | 0,21 | 1437853_x_at | NM_010882 |
| Rcsd1 | RCSD domain containing 1 | 0,21 | 1424194_at | --- |
| Ptprd | protein tyrosine phosphatase, receptor type, D | 0,21 | 1429052_at | NM_001014288 |
| Ndn | necdin | 0,21 | 1435383_x_at | NM_010882 |
| Galntl4 | UDP-N-acetyl-alpha-D-galactosamine:polypeptide N-acetylgalactosaminyltransferase-like 4 | 0,21 | 1454780_at | NM_008083 |
| Papss2 | 3'-phosphoadenosine 5'-phosphosulfate synthase 2 | 0,21 | 1434510_at | --- |
| Ndn | necdin | 0,21 | 1435382_at | NM_010882 |
| Asah3l | N-acylsphingosine amidohydrolase 3-like | 0,21 | 1451355_at | NM_178405 |
| Arhgef15 | Rho guanine nucleotide exchange factor (GEF) 15 | 0,21 | 1440358_at | NM_177566 |
| Gucy1a3 | guanylate cyclase 1, soluble, alpha 3 | 0,21 | 1434141_at | NM_021896 |
| AI465270 | expressed sequence AI465270 | 0,21 | 1438705_at | --- |
| Rapgef3 | Rap guanine nucleotide exchange factor (GEF) 3 | 0,21 | 1437012_x_at | NM_144850 |
| Sema6d | sema domain, transmembrane domain (TM), and cytoplasmic domain, (semaphorin) 6D | 0,21 | 1453055_at | NM_008458 |
| Zfhx1a | zinc finger homeobox 1a | 0,21 | 1418926_at | --- |
| 2810004A10Rik | RIKEN cDNA 2810004A10 gene | 0,21 | 1429893_at | --- |
| Tmcc3 | transmembrane and coiled coil domains 3 | 0,21 | 1434252_at | NM_021436 |
| BC054438 | cDNA sequence BC054438 | 0,21 | 1436698_x_at | NM_001001183 |
| 3732412D22Rik | RIKEN cDNA 3732412D22 gene | 0,21 | 1435106_at | NM_172753 |
| Rasgrp2 | RAS, guanyl releasing protein 2 | 0,20 | 1438933_x_at | NM_011242 |
| Sh3bp5 | SH3-domain binding protein 5 (BTK-associated) | 0,20 | 1421923_at | NM_172628 |
| Ablim3 | actin binding LIM protein family, member 3 | 0,20 | 1434013_at | NM_028765 |
| --- | Transcribed locus | 0,20 | 1445421_at | --- |
| 6230424C14Rik | RIKEN cDNA 6230424C14 gene | 0,20 | 1441972_at | --- |
| Ndn | Necdin | 0,20 | 1455792_x_at | --- |
| Cyp2d22 | cytochrome P450, family 2, subfamily d, polypeptide 22 | 0,20 | 1419040_at | NM_007823 |
| Gstm2 | glutathione S-transferase, mu 2 | 0,20 | 1416411_at | NM_144513 |
| Sox7 | SRY-box containing gene 7 | 0,20 | 1416564_at | NM_052994 |
| Adamts8 | a disintegrin-like and metalloprotease (reprolysin type) with thrombospondin type 1 motif, 8 | 0,20 | 1444174_at | NM_080435 |
| Edg6 | endothelial differentiation, G-protein-coupled receptor 6 | 0,20 | 1451024_at | NM_010103 |
| 1190002N15Rik | RIKEN cDNA 1190002N15 gene | 0,20 | 1433581_at | XM_147036 |
| Egfl7 | EGF-like domain 7 | 0,20 | 1451428_x_at | NM_178444 |
| Gimap6 | GTPase, IMAP family member 6 | 0,20 | 1427891_at | NM_008121 |
| Agpat4 | 1-acylglycerol-3-phosphate O-acyltransferase 1 (lysophosphatidic acid acyltransferase, delta) | 0,20 | 1436640_x_at |  |
| Meis1 | Myeloid ecotropic viral integration site 1 | 0,20 | 1450992_a_at | NM_029568 |
| Arhgef10 | Rho guanine nucleotide exchange factor (GEF) 10 | 0,20 | 1452302_at | NM_177566 |
| Dpyd | dihydropyrimidine dehydrogenase | 0,19 | 1427946_s_at | NM_030598 |
| Cdr2 | cerebellar degeneration-related 2 | 0,19 | 1417430_at | NM_001008424 |
| Tnrc9 | trinucleotide repeat containing 9 | 0,19 | 1436600_at | NM_172623 |
| --- | --- | 0,19 | 1438027_at | --- |
| Ldb2 | LIM domain binding 2 | 0,19 | 1439557_s_at | NM_010698 |
| Phactr1 | phosphatase and actin regulator 1 | 0,19 | 1454832_at | NM_145823 |
| Sell | selectin, lymphocyte | 0,19 | 1419481_at | NM_011346 |
| Nostrin | nitric oxide synthase trafficker | 0,19 | 1441075_at | NM_010929 |
| Gucy1b3 | guanylate cyclase 1, soluble, beta 3 | 0,19 | 1420872_at | NM_010370 |
| Pcdh19 | protocadherin 19 | 0,19 | 1437360_at | --- |
| Ncald | neurocalcin delta | 0,19 | 1417568_at | --- |
| 6430702L12 | hypothetical protein 6430702L12 | 0,19 | 1442226_at | NM_177793 |
| Reck | reversion-inducing-cysteine-rich protein with kazal motifs | 0,19 | 1450784_at | NM_173402 |
| Stxbp6 | syntaxin binding protein 6 (amisyn) | 0,19 | 1425749_at | NM_144552 |
| Slc24a3 | solute carrier family 24 (sodium/potassium/calcium exchanger), member 3 | 0,19 | 1424308_at | NM_011401 |
| Mrg1 | myeloid ecotropic viral integration site-related gene 1 | 0,19 | 1417129_a_at | NM_007641 |
| Egfl7 | EGF-like domain 7 | 0,19 | 1421335_a_at | NM_178444 |
| Kit | kit oncogene | 0,19 | 1452514_a_at | NM_013598 |
| Thbd | thrombomodulin | 0,19 | 1448529_at | NM_011587 |
| Pecam1 | platelet/endothelial cell adhesion molecule 1 | 0,19 | 1421287_a_at | NM_001005740 |
| --- | Transcribed locus | 0,18 | 1455418_at | --- |
| Pdlim3 | PDZ and LIM domain 3 | 0,18 | 1449178_at | --- |
| 5033414K04Rik | RIKEN cDNA 5033414K04 gene | 0,18 | 1436999_at | NM_026153 |
| Fgf7 | fibroblast growth factor 7 | 0,18 | 1438405_at | NM_008011 |
| Rapgef3 | Rap guanine nucleotide exchange factor (GEF) 3 | 0,18 | 1424471_at | --- |
| 1700008G05Rik | RIKEN cDNA 1700008G05 gene | 0,18 | 1431788_at | NM_025467 |
| Gm1752 | gene model 1752, (NCBI) | 0,18 | 1436941_at | XM_128908 |
| Gna14 | guanine nucleotide binding protein, alpha 14 | 0,18 | 1420385_at | NM_008137 |
| 1200009O22Rik | RIKEN cDNA 1200009O22 gene | 0,18 | 1452961_at | NM_025817 |
| Rasgrp3 | RAS, guanyl releasing protein 3 | 0,18 | 1438030_at | --- |
| Gna14 | guanine nucleotide binding protein, alpha 14 | 0,18 | 1447791_s_at | NM_001001999 |
| St8sia2 | ST8 alpha-N-acetyl-neuraminide alpha-2,8-sialyltransferase 2 | 0,18 | 1420377_at | NM_145838 |
| Prickle2 | prickle-like 2 (Drosophila) | 0,18 | 1428808_at | NM_011104 |
| Scrn1 | secernin 1 | 0,18 | 1439500_at | NM_020052 |
| 9430020K01Rik | RIKEN cDNA 9430020K01 gene | 0,18 | 1428535_at | XM_125627 |
| Satb1 | special AT-rich sequence binding protein 1 | 0,18 | 1416007_at | XM_141275 |
| 6230424C14Rik | RIKEN cDNA 6230424C14 gene | 0,18 | 1429543_at | --- |
| 1110032E23Rik | RIKEN cDNA 1110032E23 gene | 0,18 | 1416805_at | --- |
| Dpt | dermatopontin | 0,18 | 1418511_at | NM_170778 |
| Wisp2 | WNT1 inducible signaling pathway protein 2 | 0,18 | 1419015_at | NM_023653 |
| Pcdh12 | protocadherin 12 | 0,18 | 1437928_at | NM_130448 |
| Car14 | carbonic anhydrase 14 | 0,17 | 1450725_s_at | --- |
| Hspa12b | heat shock protein 12B | 0,17 | 1460371_at | NM_010494 |
| Edg3 | endothelial differentiation, sphingolipid G-protein-coupled receptor, 3 | 0,17 | 1437173_at | NM_010101 |
| BC054438 | cDNA sequence BC054438 | 0,17 | 1434621_at | NM_009738 |
| 1190002N15Rik | RIKEN cDNA 1190002N15 gene | 0,17 | 1433582_at | NM_025817 |
| Spon1 | spondin 1, (f-spondin) extracellular matrix protein | 0,17 | 1441226_at | NM_009264 |
| Tnfsf10 | tumor necrosis factor (ligand) superfamily, member 10 | 0,17 | 1459913_at | --- |
| Vtn | vitronectin | 0,17 | 1455098_a_at | NM_011707 |
| Ptprd | protein tyrosine phosphatase, receptor type, D | 0,17 | 1424886_at | NM_001014288 |
| H2-Ob | histocompatibility 2, O region beta locus | 0,17 | 1440837_at | NM_008218 |
| Eng | endoglin | 0,17 | 1432176_a_at | NM_013813 |
| Nt5dc2 | 5'-nucleotidase domain containing 2 | 0,17 | 1424882_a_at | NM_008744 |
| Pltp | phospholipid transfer protein | 0,17 | 1456424_s_at | NM_011125 |
| Ushbp1 | Usher syndrome 1C binding protein 1 | 0,17 | 1451500_at | NM_012038 |
| Pxdn | peroxidasin homolog | 0,17 | 1428259_at | NM_019444 |
| Sema6a | sema domain, transmembrane domain (TM), and cytoplasmic domain, (semaphorin) 6A | 0,17 | 1436458_at | NM_172537 |
| Aff2 | AF4/FMR2 family, member 2 | 0,17 | 1443847_x_at | --- |
| Akap2 | A kinase (PRKA) anchor protein 2 | 0,17 | 1455870_at | --- |
| Pdzd2 | PDZ domain containing 2 | 0,17 | 1435553_at | NM_008816 |
| Shank3 | SH3/ankyrin domain gene 3 | 0,17 | 1419137_at | --- |
| Tmem71 | transmembrane protein 71 | 0,17 | 1436212_at | --- |
| 4732435N03Rik | RIKEN cDNA 4732435N03 gene | 0,17 | 1452366_at | --- |
| Mapt | microtubule-associated protein tau | 0,17 | 1445634_at | NM_010838 |
| Arhgap20 | Rho GTPase activating protein 20 | 0,17 | 1429918_at | NM_172751 |
| Kitl | kit ligand | 0,16 | 1415855_at | NM_010636 |
| Tm4sf1 | transmembrane 4 superfamily member 1 | 0,16 | 1439925_at | NM_178874 |
| Ptprb | protein tyrosine phosphatase, receptor type, B | 0,16 | 1436367_at | NM_029928 |
| Myh10 | Myosin, heavy polypeptide 10, non-muscle | 0,16 | 1452740_at | NM_144557 |
| Dnajb6 | DnaJ (Hsp40) homolog, subfamily B, member 6 | 0,16 | 1435701_at | NM_007876 |
| Dgkg | diacylglycerol kinase, gamma | 0,16 | 1431167_at | --- |
| Plod2 | procollagen lysine, 2-oxoglutarate 5-dioxygenase 2 | 0,16 | 1416686_at | NM_011125 |
| Gata2 | GATA binding protein 2 | 0,16 | 1450333_a_at | NM_008090 |
| Pcdh1 | protocadherin 1 | 0,16 | 1452294_at | NM_017378 |
| Agtr1a | angiotensin II receptor, type 1a | 0,16 | 1436739_at | --- |
| Ppp1r16b | protein phosphatase 1, regulatory (inhibitor) subunit 16B | 0,16 | 1455080_at | XM_128308 |
| Ldb2 | LIM domain binding 2 | 0,16 | 1456786_at | NM_010698 |
| Tspan13 | tetraspanin 13 | 0,16 | 1460239_at | NM_025359 |
| C1qtnf2 | C1q and tumor necrosis factor related protein 2 | 0,16 | 1431079_at | NM_028981 |
| Egfl7 | EGF-like domain 7 | 0,16 | 1451427_a_at | --- |
| Grap | GRB2-related adaptor protein | 0,16 | 1429387_at | NM_008183 |
| Rgs12 | regulator of G-protein signaling 12 | 0,16 | 1453129_a_at | NM_022881 |
| Cd97 | CD97 antigen | 0,16 | 1418394_a_at | NM_009868 |
| Klf2 | Kruppel-like factor 2 (lung) | 0,16 | 1448890_at | NM_008457 |
| Pcsk5 | proprotein convertase subtilisin/kexin type 5 | 0,16 | 1451406_a_at | XM_129214 |
| Tnik /// LOC665113 /// LOC674765 | TRAF2 and NCK interacting kinase | 0,16 | 1455256_at | NM_009393 |
| Slc36a2 | solute carrier family 36 (proton/amino acid symporter), member 2 | 0,16 | 1436521_at | NM_172479 |
| Pitpnc1 | phosphatidylinositol transfer protein, cytoplasmic 1 | 0,16 | 1428878_a_at | NM_145823 |
| BC028528 | CDNA sequence BC028528 | 0,16 | 1427996_at | NM_153513 |
| Sema3e | sema domain, immunoglobulin domain (Ig), short basic domain, secreted, (semaphorin) 3E | 0,16 | 1427673_a_at | NM_011348 |
| Stk23 | serine/threonine kinase 23 | 0,16 | 1447806_s_at | NM_025285 |
| Vsnl1 | visinin-like 1 | 0,16 | 1450055_at | NM_012038 |
| Gata2 | GATA binding protein 2 | 0,16 | 1428816_a_at | NM_008091 |
| Pcdh18 | protocadherin 18 | 0,16 | 1422890_at | NM_130448 |
| Sdpr | serum deprivation response | 0,16 | 1416778_at | NM_138741 |
| BC020535 | cDNA sequence BC020535 | 0,15 | 1435600_s_at | NM_153513 |
| Rasal2 | RAS protein activator like 2 | 0,15 | 1436910_at | NM_011245 |
| Rtn1 | reticulon 1 | 0,15 | 1429761_at | NM_025393 |
| Tmeff2 | transmembrane protein with EGF-like and two follistatin-like domains 2 | 0,15 | 1419073_at | --- |
| Ms4a1 | membrane-spanning 4-domains, subfamily A, member 1 | 0,15 | 1423226_at | NM_021718 |
| AI661384 | expressed sequence AI661384 | 0,15 | 1435710_at | NM_031185 |
| Admr | adrenomedullin receptor | 0,15 | 1418554_at | NM_001008533 |
| Bmp6 | bone morphogenetic protein 6 | 0,15 | 1459947_at | --- |
| Cdo1 | cysteine dioxygenase 1, cytosolic | 0,15 | 1448842_at | NM_007672 |
| Pcsk5 | proprotein convertase subtilisin/kexin type 5 | 0,15 | 1437339_s_at | XM_355911 |
| Tie1 | tyrosine kinase receptor 1 | 0,15 | 1416238_at | NM_008536 |
| Atp1a2 | ATPase, Na+/K+ transporting, alpha 2 polypeptide | 0,15 | 1434893_at | NM_178405 |
| 3021401C12Rik | RIKEN cDNA 3021401C12 gene | 0,15 | 1453782_at | NM_001001980 |
| Tmeff1 | transmembrane protein with EGF-like and two follistatin-like domains 1 | 0,15 | 1426649_at | NM_019790 |
| Dach1 | dachshund 1 (Drosophila) | 0,15 | 1433743_at | NM_026993 |
| Stard9 /// LOC668856 /// LOC670354 |  | 0,15 | 1436324_at | NM_019684 |
| Ga17 | Dendritic cell protein GA17 | 0,15 | 1457587_at | NM_173739 |
| Ddc | dopa decarboxylase | 0,15 | 1426215_at | NM_010043 |
| Gpr126 | G protein-coupled receptor 126 | 0,15 | 1437408_at | NM_001002268 |
| Ldb2 | RIKEN cDNA E030026E10 gene | 0,15 | 1421101_a_at | NM_144862 |
| Rgs18 | regulator of G-protein signaling 18 | 0,15 | 1449856_at | NM_022881 |
| Pcdh18 | protocadherin 18 | 0,15 | 1422889_at | XM_205287 |
| Sema3e | sema domain, immunoglobulin domain (Ig), short basic domain, secreted, (semaphorin) 3E | 0,15 | 1419717_at | NM_018744 |
| Hoxa5 | homeo box A5 | 0,15 | 1448926_at | NM_008268 |
| Pitpnc1 | phosphatidylinositol transfer protein, cytoplasmic 1 | 0,15 | 1452939_a_at | NM_145823 |
| Notch4 | Notch gene homolog 4 (Drosophila) | 0,15 | 1449146_at | NM_010942 |
| She | src homology 2 domain-containing transforming protein E | 0,15 | 1438227_at | NM_025807 |
| 1110065P19Rik /// 2310040A07Rik | RIKEN cDNA 1110065P19 gene /// RIKEN cDNA 2310040A07 gene | 0,15 | 1452893_s_at | XM_147036 |
| 2210023G05Rik | RIKEN cDNA 2210023G05 gene | 0,14 | 1424968_at | --- |
| Epb4.1l3 | erythrocyte protein band 4.1-like 3 | 0,14 | 1419062_at | NM_007950 |
| Slc43a3 | solute carrier family 43, member 3 | 0,14 | 1422788_at | NM_017394 |
| Edg3 | endothelial differentiation, sphingolipid G-protein-coupled receptor, 3 | 0,14 | 1438658_a_at | NM_010102 |
| Ramp2 | receptor (calcitonin) activity modifying protein 2 | 0,14 | 1418187_at | NM_144850 |
| Tspan18 | tetraspanin 18 | 0,14 | 1442174_at | NM_019634 |
| Fxyd1 | FXYD domain-containing ion transport regulator 1 | 0,14 | 1421374_a_at | --- |
| Kdr | kinase insert domain protein receptor | 0,14 | 1449379_at | --- |
| BB114106 | expressed sequence BB114106 | 0,14 | 1439527_at | NM_145536 |
| Rgs18 | regulator of G-protein signaling 18 | 0,14 | 1420398_at | --- |
| Unc45b | unc-45 homolog B | 0,14 | 1436939_at | NM_178924 |
| Hhip | Hedgehog-interacting protein | 0,14 | 1455277_at | NM_010453 |
| Gpr126 | G protein-coupled receptor 126 | 0,14 | 1437409_s_at | NM_027817 |
| Gucy1a3 | guanylate cyclase 1, soluble, alpha 3 | 0,14 | 1420534_at | NM_017469 |
| Fat3 | FAT tumor suppressor homolog 3 | 0,14 | 1457589_at | NM_021564 |
| Stmn2 | stathmin-like 2 | 0,14 | 1423281_at | NM_144552 |
| 6330500D04Rik | RIKEN cDNA 6330500D04 gene | 0,14 | 1460555_at | --- |
| Tmtc2 | transmembrane and tetratricopeptide repeat containing 2 | 0,14 | 1429809_at | NM_009425 |
| Upk1b | uroplakin 1B | 0,14 | 1455464_x_at | --- |
| Gp1bb | glycoprotein Ib, beta polypeptide | 0,14 | 1422977_at | NM_018762 |
| Abi3bp | ABI gene family, member 3 (NESH) binding protein | 0,14 | 1427054_s_at | --- |
| Ccdc85a | coiled-coil domain containing 85A | 0,14 | 1445204_at | NM_009844 |
| Gja5 | gap junction membrane channel protein alpha 5 | 0,14 | 1429101_at | --- |
| Diras2 | DIRAS family, GTP-binding RAS-like 2 | 0,14 | 1455436_at | NM_183312 |
| Scn3b | sodium channel, voltage-gated, type III, beta | 0,14 | 1435767_at | NM_009135 |
| Cd19 | CD19 antigen | 0,14 | 1450570_a_at | NM_011925 |
| Msln | mesothelin | 0,14 | 1460238_at | NM_175260 |
| Tspan13 | tetraspanin 13 | 0,14 | 1418643_at | --- |
| Mamdc2 | MAM domain containing 2 | 0,14 | 1453152_at | NM_010838 |
| Slc2a3 | solute carrier family 2 (facilitated glucose transporter), member 3 | 0,13 | 1437052_s_at | NM_153170 |
| LOC623121 | similar to Interferon-activatable protein 203 | 0,13 | 1437636_at | --- |
| Xlkd1 | extra cellular link domain-containing 1 | 0,13 | 1429379_at | NM_053247 |
| Wfdc6a | WAP four-disulfide core domain 6A | 0,13 | 1457766_at | NM_016873 |
| Pitpnc1 | phosphatidylinositol transfer protein, cytoplasmic 1 | 0,13 | 1453750_x_at | NM_145823 |
| Sgip1 | SH3-domain GRB2-like (endophilin) interacting protein 1 | 0,13 | 1425180_at | NM_144906 |
| 2700055K07Rik | RIKEN cDNA 2700055K07 gene | 0,13 | 1416713_at | NM_027265 |
| Treml4 | triggering receptor expressed on myeloid cells-like 4 | 0,13 | 1460014_at | NM_025359 |
| Pltp | phospholipid transfer protein | 0,13 | 1417963_at | NM_028199 |
| Atp1a2 | ATPase, Na+/K+ transporting, alpha 2 polypeptide | 0,13 | 1452308_a_at | NM_178405 |
| Plcl1 | phospholipase C-like 1 | 0,13 | 1445723_at | XM_483915 |
| Centd3 | centaurin, delta 3 | 0,13 | 1451282_at | NM_139206 |
| Tspan7 | tetraspanin 7 | 0,13 | 1417502_at | NM_019634 |
| 1200009O22Rik | RIKEN cDNA 1200009O22 gene | 0,13 | 1428922_at | XM_133706 |
| Lphn3 | latrophilin 3 | 0,13 | 1447551_x_at | NM_023624 |
| Efemp1 | epidermal growth factor-containing fibulin-like extracellular matrix protein 1 | 0,13 | 1427183_at | NM_010111 |
| Cyt1 | cytokine like 1 | 0,13 | 1456793_at | --- |
| Lims2 | LIM and senescent cell antigen like domains 2 | 0,13 | 1424408_at | NM_010720 |
| 2310016C08Rik | RIKEN cDNA 2310016C08 gene | 0,13 | 1421031_a_at | --- |
| D330027H18Rik | RIKEN cDNA D330027H18 gene | 0,12 | 1439728_at | --- |
| Myrip | myosin VIIA and Rab interacting protein | 0,12 | 1460601_at | NM_134094 |
| Gpc3 | RIKEN cDNA D230050J18 gene | 0,12 | 1450990_at | NM_011821 |
| Scn7a | sodium channel, voltage-gated, type VII, alpha | 0,12 | 1436044_at | NM_009135 |
| Pde9a | phosphodiesterase 9A | 0,12 | 1449403_at | NM_011057 |
| Angpt1 | angiopoietin 1 | 0,12 | 1439066_at | NM_013468 |
| Ddah1 | dimethylarginine dimethylaminohydrolase 1 | 0,12 | 1429298_at | NM_026993 |
| LOC670044 | similar to Mothers against decapentaplegic homolog 6 | 0,12 | 1422771_at | NM_198702 |
| Dscr6 | Down syndrome critical region homolog 6 (human) | 0,12 | 1420459_at | --- |
| Ptprb | protein tyrosine phosphatase, receptor type, B | 0,12 | 1427486_at | NM_001014288 |
| Pitpnc1 | phosphatidylinositol transfer protein, cytoplasmic 1 | 0,12 | 1452940_x_at | NM_145823 |
| Acvrl1 | Activin A receptor, type II-like 1 | 0,12 | 1435825_at | NM_009612 |
| Spock2 | sparc/osteonectin, cwcv and kazal-like domains proteoglycan 2 | 0,12 | 1435026_at | NM_145584 |
| Cdh5 | cadherin 5 | 0,12 | 1433956_at | NM_033037 |
| D430019H16Rik | RIKEN cDNA D430019H16 gene | 0,12 | 1455447_at | XM_485742 |
| Ankrd1 | ankyrin repeat domain 1 (cardiac muscle) | 0,12 | 1420991_at | NM_013468 |
| Klf12 | RIKEN cDNA C130057G02 gene | 0,12 | 1455521_at | NM_010636 |
| Ddah1 | dimethylarginine dimethylaminohydrolase 1 | 0,12 | 1455400_at | --- |
| Lrat | lecithin-retinol acyltransferase (phosphatidylcholine-retinol-O-acyltransferase) | 0,12 | 1444487_at | NM_010733 |
| 1500016O10Rik | RIKEN cDNA 1500016O10 gene | 0,12 | 1438641_x_at | NM_029310 |
| Hey1 | hairy/enhancer-of-split related with YRPW motif 1 | 0,12 | 1415999_at | NM_020259 |
| Sell | selectin, lymphocyte | 0,12 | 1419480_at | NM_013657 |
| Cyp4b1 | cytochrome P450, family 4, subfamily b, polypeptide 1 | 0,11 | 1416194_at | XM_132070 |
| Hoxb5 | homeo box B5 | 0,11 | 1418415_at | NM_174998 |
| Tubb4 | tubulin, beta 4 | 0,11 | 1423221_at | --- |
| Ecm2 | extracellular matrix protein 2, female organ and adipocyte specific | 0,11 | 1440096_at | NM_010101 |
| Cox4i2 | cytochrome c oxidase subunit IV isoform 2 | 0,11 | 1421373_at | NM_007751 |
| Pitpnc1 | Phosphatidylinositol transfer protein, cytoplasmic 1 | 0,11 | 1455204_at | NM_145823 |
| Upk1b | uroplakin 1B | 0,11 | 1435831_at | NM_175309 |
| Centd3 | centaurin, delta 3 | 0,11 | 1419833_s_at | NM_007697 |
| Scn7a | sodium channel, voltage-gated, type VII, alpha | 0,11 | 1436043_at | NM_027268 |
| Cacna1d | calcium channel, voltage-dependent, L type, alpha 1D subunit | 0,11 | 1427974_s_at | NM_018782 |
| Ankrd1 | ankyrin repeat domain 1 (cardiac muscle) | 0,11 | 1420992_at | NM_024204 |
| F730031O20Rik | RIKEN cDNA F730031O20 gene | 0,11 | 1444426_at | --- |
| Tek | endothelial-specific receptor tyrosine kinase | 0,11 | 1418788_at | NM_009378 |
| Xlkd1 | extra cellular link domain-containing 1 | 0,11 | 1453128_at | NM_011546 |
| Ankrd38 | KN motif and ankyrin repeat domains 38 | 0,11 | 1436425_at | NM_172872 |
| Rasgrp2 | RAS, guanyl releasing protein 2 | 0,11 | 1417804_at | NM_207246 |
| 2410004I01Rik | RIKEN cDNA 2410004I01 gene | 0,11 | 1436612_at | NM_026481 |
| 2210419I08Rik | RIKEN cDNA 2210419I08 gene | 0,11 | 1429637_at | NM_023516 |
| Arhgef15 | Rho guanine nucleotide exchange factor (GEF) 15 | 0,11 | 1455522_at | NM_030066 |
| Rspo1 | R-spondin homolog | 0,11 | 1449319_at | NM_001007596 |
| 9030224M15Rik | RIKEN cDNA 9030224M15 gene | 0,11 | 1439689_at | NM_145562 |
| Icam2 | intercellular adhesion molecule 2 | 0,11 | 1448862_at | XM_133956 |
| Foxf1a | forkhead box F1a | 0,11 | 1434939_at | NM_008046 |
| Pitpnc1 | phosphatidylinositol transfer protein, cytoplasmic 1 | 0,11 | 1435066_at | NM_145823 |
| Col13a1 | procollagen, type XIII, alpha 1 | 0,11 | 1422866_at | NM_053091 |
| Clec14a | C-type lectin domain family 14, member a | 0,10 | 1419467_at | NM_025809 |
| Sdpr | serum deprivation response | 0,10 | 1416779_at | NM_138741 |
| Gzma | granzyme A | 0,10 | 1417898_a_at | --- |
| Gata3 | GATA binding protein 3 | 0,10 | 1448886_at | NM_008115 |
| Tbx3 | T-box 3 | 0,10 | 1448029_at | NM_011535 |
| Dnahc8 | dynein, axonemal, heavy chain 8 | 0,10 | 1424936_a_at | NM_011847 |
| Tspan7 | tetraspanin 7 | 0,10 | 1448737_at | NM_009451 |
| BC030477 | cDNA sequence BC030477 | 0,10 | 1455845_at | NM_153803 |
| BC028528 | CDNA sequence BC028528 | 0,10 | 1445301_at | NM_177618 |
| Hba-a1 | hemoglobin alpha, adult chain 1 | 0,10 | 1417714_x_at | NM_010423 |
| Aff3 | AF4/FMR2 family, member 3 | 0,10 | 1441172_at | NM_026644 |
| G0s2 | G0/G1 switch gene 2 | 0,10 | 1448700_at | NM_145380 |
| Popdc3 | popeye domain containing 3 | 0,10 | 1423856_at | NM_080555 |
| Cldn5 | claudin 5 | 0,10 | 1417839_at | NM_025809 |
| Atp1a2 | ATPase, Na+/K+ transporting, alpha 2 polypeptide | 0,10 | 1443823_s_at | NM_178405 |
| Ppp1r14a | protein phosphatase 1, regulatory (inhibitor) subunit 14A | 0,10 | 1418086_at | NM_153089 |
| Igh-1a | immunoglobulin heavy chain 1a (serum IgG2a) | 0,10 | 1455530_at | NM_011829 |
| Acoxl | acyl-Coenzyme A oxidase-like | 0,10 | 1460470_at | NM_009608 |
| D11Bwg0517e | DNA segment, Chr 11, Brigham & Women's Genetics 0517 expressed | 0,10 | 1436450_at | XM_149840 |
| AA407270 | expressed sequence AA407270 | 0,10 | 1455180_at | NM_175503 |
| Mcc | mutated in colorectal cancers | 0,10 | 1438081_at | NM_010789 |
| Stxbp6 | syntaxin binding protein 6 (amisyn) | 0,10 | 1435396_at | NM_011535 |
| Sgip1 | SH3-domain GRB2-like (endophilin) interacting protein 1 | 0,10 | 1425181_at | NM_011894 |
| BC038479 | cDNA sequence BC038479 | 0,10 | 1433727_at | NM_001001183 |
| Gfra2 | glial cell line derived neurotrophic factor family receptor alpha 2 | 0,10 | 1433716_x_at | NM_010284 |
| 5730557B15Rik | RIKEN cDNA 5730557B15 gene | 0,10 | 1453287_at | --- |
| Tbx3 | T-box 3 | 0,09 | 1437479_x_at | NM_011545 |
| Prx | periaxin | 0,09 | 1423292_a_at | NM_008966 |
| Art3 | ADP-ribosyltransferase 3 | 0,09 | 1452474_a_at | --- |
| Ptgfr | prostaglandin F receptor | 0,09 | 1446331_at | NM_008970 |
| --- | 0 day neonate lung cDNA, RIKEN full-length enriched library, clone:E030034L15 product:unclassifiable, full insert sequence | 0,09 | 1443137_at | --- |
| Clec14a | C-type lectin domain family 14, member a | 0,09 | 1419468_at | NM_175526 |
| Chrdl1 | chordin-like 1 | 0,09 | 1456722_at | NM_023850 |
| Vtn | vitronectin | 0,09 | 1420484_a_at | --- |
| Clec1a | C-type lectin domain family 1, member a | 0,09 | 1456318_at | NM_175526 |
| Gpm6a | glycoprotein m6a | 0,09 | 1426442_at | NM_153581 |
| Aard | alanine and arginine rich domain containing protein | 0,08 | 1434528_at | NM_030239 |
| Itga8 | integrin alpha 8 | 0,08 | 1427489_at | NM_001001309 |
| Pitpnc1 | phosphatidylinositol transfer protein, cytoplasmic 1 | 0,08 | 1431074_a_at | NM_153179 |
| Klf12 | Kruppel-like factor 12 | 0,08 | 1439847_s_at | NM_008452 |
| Acvrl1 | activin A receptor, type II-like 1 | 0,08 | 1451604_a_at | NM_013906 |
| Chst1 | carbohydrate (keratan sulfate Gal-6) sulfotransferase 1 | 0,08 | 1449147_at | NM_009897 |
| Abcg3 | ATP-binding cassette, sub-family G (WHITE), member 3 | 0,08 | 1421168_at | --- |
| Calcrl | calcitonin receptor-like | 0,08 | 1418489_a_at | NM_011797 |
| Lrrn3 | leucine rich repeat protein 3, neuronal | 0,08 | 1434539_at |  |
| 1110050K14Rik | RIKEN cDNA 1110050K14 gene | 0,08 | 1430012_at | --- |
| Gap43 | Growth associated protein 43 | 0,08 | 1423537_at | NM_008090 |
| Akap12 | A kinase (PRKA) anchor protein (gravin) 12 | 0,08 | 1419706_a_at | NM_009649 |
| Kif26a /// LOC668303 /// LOC676226 | kinesin family member 26A | 0,08 | 1447933_at | NM_021099 |
| Emr4 | EGF-like module containing, mucin-like, hormone receptor-like sequence 4 | 0,07 | 1451563_at | NM_007932 |
| Clec1a | C-type lectin domain family 1, member a | 0,07 | 1460039_at | NM_011606 |
| Tmem46 | transmembrane protein 46 | 0,07 | 1423852_at | --- |
| Dscr1l1 | Down syndrome critical region gene 1-like 1 | 0,07 | 1421425_a_at | NM_133229 |
| Wnt2 | wingless-related MMTV integration site 2 | 0,07 | 1449425_at | NM_053247 |
| Alas2 | aminolevulinic acid synthase 2, erythroid | 0,07 | 1451675_a_at | NM_013467 |
| Lipg | lipase, endothelial | 0,07 | 1450188_s_at | NM_010720 |
| Prickle1 | prickle like 1 (Drosophila) | 0,07 | 1452249_at | XM_144905 |
| Sema3g | sema domain, immunoglobulin domain (Ig), short basic domain, secreted, (semaphorin) 3G | 0,07 | 1435361_at | --- |
| --- | Transcribed locus | 0,07 | 1444480_at | --- |
| Sdpr | serum deprivation response | 0,07 | 1443832_s_at | NM_011346 |
| Bche | butyrylcholinesterase | 0,07 | 1436098_at | NM_007556 |
| Edil3 | EGF-like repeats and discoidin I-like domains 3 | 0,07 | 1433474_at | NM_007904 |
| Ifitm6 | interferon induced transmembrane protein 6 | 0,07 | 1440865_at | NM_008342 |
| Atp1a2 | ATPase, Na+/K+ transporting, alpha 2 polypeptide | 0,07 | 1427465_at | NM_080467 |
| Lipg | lipase, endothelial | 0,07 | 1421262_at | NM_008505 |
| Sox11 | SRY-box containing gene 11 | 0,07 | 1453125_at | NM_009234 |
| Sox11 | SRY-box containing gene 11 | 0,07 | 1429372_at | NM_009234 |
| Fgfr4 | fibroblast growth factor receptor 4 | 0,07 | 1418596_at | NM_010216 |
| Tmcc2 | transmembrane and coiled-coil domains 2 | 0,06 | 1428108_x_at | NM_178874 |
| Sox11 | SRY-box containing gene 11 | 0,06 | 1429051_s_at | NM_009234 |
| Itga8 | integrin alpha 8 | 0,06 | 1454966_at | NM_010582 |
| Dpep1 | dipeptidase 1 (renal) | 0,06 | 1419674_a_at | NM_007876 |
| A730046J16 | hypothetical protein A730046J16 | 0,06 | 1433939_at | --- |
| Mapt | microtubule-associated protein tau | 0,06 | 1424718_at | NM_010838 |
| 2810484G07Rik | RIKEN cDNA 2810484G07 gene | 0,06 | 1444214_at | --- |
| Actc1 | actin, alpha, cardiac | 0,06 | 1415927_at | NM_009612 |
| Hpgd | Hydroxyprostaglandin dehydrogenase 15 (NAD) | 0,06 | 1419906_at | --- |
| Tmem100 | transmembrane protein 100 | 0,06 | 1446625_at | --- |
| Serpina3c | serine (or cysteine) proteinase inhibitor, clade A, member 3C | 0,06 | 1421564_at | --- |
| --- | 16 days neonate cerebellum cDNA, RIKEN full-length enriched library, clone:9630041I08 product:unclassifiable, full insert sequence | 0,06 | 1439732_at | --- |
| Tcf21 | Transcription factor 21 | 0,06 | 1417447_at | NM_013690 |
| Clec3b | C-type lectin domain family 3, member b | 0,06 | 1449466_at | NM_007731 |
| Mapt | microtubule-associated protein tau | 0,06 | 1424719_a_at | NM_010838 |
| Sh3tc2 | SH3 domain and tetratricopeptide repeats 2 | 0,05 | 1456020_at | NM_021423 |
| Mapt | Microtubule-associated protein tau | 0,05 | 1455028_at | XM_140309 |
| Prkg2 | protein kinase, cGMP-dependent, type II | 0,05 | 1435460_at | NM_008926 |
| Pcolce2 | procollagen C-endopeptidase enhancer 2 | 0,05 | 1451527_at | XM_129214 |
| Prkg2 | Protein kinase, cGMP-dependent, type II | 0,05 | 1435162_at | NM_019412 |
| 1110018M03Rik | RIKEN cDNA 1110018M03 gene | 0,05 | 1419376_at | NM_133187 |
| Gpm6a | glycoprotein m6a | 0,05 | 1456741_s_at | NM_001002268 |
| Vsnl1 | visinin-like 1 | 0,05 | 1420955_at | NM_011707 |
| Gm1337 | gene model 1337 | 0,05 | 1443287_at | XM_156281 |
| Fmo1 | flavin containing monooxygenase 1 | 0,05 | 1417429_at | NM_010426 |
| Adcy8 | adenylate cyclase 8 | 0,05 | 1418754_at | NM_007412 |
| Mfap4 | microfibrillar-associated protein 4 | 0,05 | 1424010_at | NM_010825 |
| Tmcc2 | transmembrane and coiled-coil domains 2 | 0,05 | 1452666_a_at | NM_172051 |
| 9430073N08Rik | RIKEN cDNA 9430073N08 gene | 0,05 | 1453496_at | XM_194000 |
| 2900001G08Rik | RIKEN cDNA 2900001G08 gene | 0,05 | 1430642_at | --- |
| Igfbp2 | insulin-like growth factor binding protein 2 | 0,05 | 1454159_a_at | XM_484178 |
| Slc7a10 | solute carrier family 7 (cationic amino acid transporter, y+ system), member 10 | 0,04 | 1421093_at | NM_009234 |
| Hpgd | hydroxyprostaglandin dehydrogenase 15 (NAD) | 0,04 | 1419905_s_at | --- |
| Dpep1 | Dipeptidase 1 (renal) | 0,04 | 1435943_at | NM_019759 |
| Figf | C-fos induced growth factor | 0,04 | 1438953_at | --- |
| Ednrb | Endothelin receptor type B | 0,04 | 1437347_at | NM_146015 |
| Slc38a5 | solute carrier family 38, member 5 | 0,04 | 1454622_at | NM_021398 |
| Tmem100 | transmembrane protein 100 | 0,04 | 1449533_at | NM_145463 |
| Figf | C-fos induced growth factor | 0,04 | 1438954_x_at | --- |
| Hpcal4 | hippocalcin-like 4 | 0,04 | 1433987_at | NM_008278 |
| Figf | c-fos induced growth factor | 0,03 | 1449528_at | NM_010231 |
| Scube2 | signal peptide, CUB domain, EGF-like 2 | 0,03 | 1453486_a_at | NM_138741 |
| Bmp6 | bone morphogenetic protein 6 | 0,03 | 1450759_at | NM_001017525 |
| Pcdha4 /// Pcdha6 /// Pcdha7 |  | 0,03 | 1420798_s_at | NM_029620 |
| Sox11 | SRY-box containing gene 1 | 0,03 | 1453002_at | NM_009235 |
| Cox8b | cytochrome c oxidase, subunit VIIIb | 0,03 | 1449218_at | NM_139001 |
| Sox11 | SRY-box containing gene 2 | 0,03 | 1431225_at | NM_009234 |
| Ogn | osteoglycin | 0,03 | 1419663_at | NM_008760 |
| Cxcl7 | chemokine (C-X-C motif) ligand 7 | 0,03 | 1418480_at | NM_019823 |
| C030019F02Rik | RIKEN cDNA C030019F02 gene | 0,03 | 1422596_at | --- |
| Ogn | osteoglycin | 0,03 | 1419662_at | NM_008768 |
| Amph | Amphiphysin | 0,03 | 1427044_a_at | NM_009640 |
| --- | Adult male olfactory brain cDNA, RIKEN full-length enriched library, clone:6430530M09 product:unclassifiable, full insert sequence | 0,02 | 1460061_at | --- |
| Adrb3 | adrenergic receptor, beta 3 | 0,02 | 1455918_at | NM_007421 |
| Scn3a | sodium channel, voltage-gated, type III, alpha | 0,02 | 1439204_at | NM_153522 |
| Upk3b | uroplakin 3B | 0,01 | 1454881_s_at | NM_181418 |
| Sox11 | SRY-box containing gene 11 | 0,01 | 1436790_a_at | NM_011439 |
