# Supplementary material for: Cancer Genomics Identifies Regulatory Gene Networks Associated with the Transition from Dysplasia to Advanced Lung Adenocarcinomas Induced by c-Raf-1
Source: PLoS One. 2009 Oct 8;4(10):e7315. doi: 10.1371/journal.pone.0007315 (PMC2754338; doi:10.1371/journal.pone.0007315)
Supplement: Text S1 — Description of gene regulated networks associated in dysplasia. (0.04 MB DOC) [file pone.0007315.s009.doc]

**Gene regulatory networks associated in dysplasia**

The Zn-finger protein Hnf4α (hepatocyte nuclear factor 4-alpha) was identified as a key molecule (Figure S5). Its regulation was confirmed by immunohistochemistry. This protein plays an essential role in regulation of enzymes which are involved in various metabolic processes e.g. lipid metabolism, RNA modification, regulation of an epithelial phenotype and of different signaling pathways. Notably, Hnf4α controls the expression of other transcription factors as well such as Hnf1 (hepatocyte nuclear factor 1) and we observed the strongly induced Pcbd1 (pterin 4 alpha carbinolamine dehydratase/dimerization cofactor of hepatocyte nuclear factor 1 alpha (TCF1) 1) as a direct target of Hnf4α. Pcbd1 acts as a cofactor for Hnf1 and stabilizes the dimeric Hnf1 complex [1].

Several additional direct target of Hnf4α such as Gsta4 (glutathione S-transferase A4), Cyp1b1 (Cytochrome P450 1B1), Apoa1 (apolipoprotein A-1) are depicted in this network. As cofactor of Lcat (lecithin-cholesterin-acetyltransferase) Apoa1 plays a role in the formation of cholesterol esters in plasma. This cholesterol efflux involves the binding of Apoa1 to the plasma membrane and requires cellular ATP-binding cassette transporter (ABCA1) activity. Moreover, Apoa1 activates cellular cAMP signalling through the ABCA1 transporter [2]. In this context we found Adcyap1 (adenylate cyclase activating polypeptide 1) to be regulated, which has a crucial role in energy metabolism including lipid metabolism [3]. A further direct target of Hnf4α in this network is Afp (alpha fetoprotein). Alpha-fetoprotein is a cancer associated fetal glycoprotein and high serum concentration of this protein is a useful marker for certain malignancies such as hepatocellular carcinoma. It has been reported that some primary lung cancers display elevated Afp [4]. Another direct target of Hnf4α is Hepsin (HPN), a type II transmembrane serine protease. Recently, it was reported that in a prostate cancer mouse model, hepsin up-regulation in tumor tissue promotes progression and metastasis [5]. Arg2 (arginase 2) is a further molecule regulated by Hnf4α. It was shown that Arg2 is expressed in lung cancer with Arg2 expression being particularly high in aggressive histological phenotypes [6]. A further direct target of Hnf4α is Lad1 (ladinin 1). Ladinin is an anchoring filament protein of basement membrane and may contribute to the stability of the association of the epithelial layers with the underlying mesenchyme [7].

A down stream target of Hnf4α we identified Rgs16 (regulator of G-protein signalling 16). Overexpression of RGS16 inhibits G protein-coupled mitogenic signal transduction and activation of the mitogen-activated protein kinase (MAPK) signalling cascade [8]. Clusterin was reported to be overexpressed in several human cancers. It could be demonstrated that clusterin inhibited apoptosis by interfering with BAX activation in mitochondria [9].

**The second network** of dysplasia shows target molecules by Hnf4α e.g. Foxa3, which is involved in glucose metabolism (Figure S5). In this network, only few members of Tgf-β are directly or indirectly regulated. That includes claudin 4, Prss22 (protease, serine, 22), Sdcbp2 (syndecan binding protein 2), CD177, Kcnk2 (potassium channel, subfamily K, member 2) and Fut2 (fucosyltransferase 2). Regulation of claudin 4 (CLDN4) is notable. This tight junction protein has been found to be overexpressed in a wide variety of cancers. Tight junction proteins are apically located to maintain cell polarity and to control paracellular permeability. Prss22 (protease, serine, 22), i.e. a direct target of Tgf-β was regulated. This protease is expressed in the airways probably in a developmentally regulated manner [10]. A further indirect target of Tgf-β is Sdcbp2 (syndecan binding protein 2) which binds to phosphatidylinositol 4, 5-bisphosphate (PIP2) via its PDZ domains. Depletion of syntenin-2 disrupts the nuclear speckles-PIP2 pattern and affects cell survival and cell division [11]. Recently it was reported that the syndecan binding protein 2 is down regulated in Pten null mouse embryonic fibroblast cells [12]. CD177 is a further regulated gene of this network and is downstream of the granulocyte colony stimulating factor 3 (CSF3). Cd177 is a cell surface glycoprotein. The exact function is not yet known but it has been suggested that this protein is involved in severe antibody-dependent diseases like transfusion-related acute lung injury [13]. A further member of this network is the potassium ion channel, subfamily K, member 2 (Kcnk2) which selectively passes K ions across membranes. It was reported that this endothelial K+ channel alters the actin network architecture [14] but until now this ion channel was not associated with tumors. The Fam167A (family with sequence similarity 167, member A) is a hypothetical gene which is highly expressed in lung tissue [15]. A further member of dysplasia network 2 is Fut2 (fucosyltransferase 2) that is involved in carbohydrate metabolism by transfer of a fucosyl group to an acceptor molecule, typically another carbohydrate or a lipid. Fucosylated oligosaccharides have been implicated in multiple cell-cell interactions in differentiation, development and malignancy [16].

Finally, in dysplasia **network 3** only up-regulated genes can be found (Figure S5). Central to this network is Tp53 (tumor protein 53) and its major partners. A direct target of Tp53 is Psrc1 (proline/serine-rich coiled-coil 1) whereas Klc3 (kinesin light chain 3) is an indirect target of Tp53 and part of the kinesin light chain gene family. Kinesines consists of two heavy and two light chains and function as molecular transporters via the microtubule. Kinesins play an essential role when it comes to bipolar mitotic spindle formation. Therefore research on targeted cancer therapies focused arround kinesin inhibitors [17]. Likewise, Hecw1 (HECT, C2 and WW domain containing E3 ubiquitin protein ligase 1) an ubiquitin ligase interacts with Tp53. There are suggestions that Hecw1 promotes induction of cisplatin mediated apoptosis in cancerous cells with functional Tp53 [18]. Like Hecw1, Rnf128 (ring finger protein 128) has an ubiquitin ligase activity. The gene product is a transmembrane protein with a ring zinc-finger motif localized in the endocytic pathway.

Next to Tp53 additional transcription factors, e.g. Etv4 and Foxp2 are depicted. Etv4 (ets variant 4) is a member of the Ets domain transcription factors and involved in oncogenesis, cancer invasiveness and metastasis. It is postulated that Etv4 provides positive regulation to induced cell apoptosis [19], while Foxp2 (forkhead box P2) belongs to the Foxp subfamily of winged-helix transcription factors and is expressed in fetal and adult brain. It is reported that forkhead proteins are important for cell cycle regulation, embryonic development and oncogenesis.

A further indirect regulated gene in this network is the proto-oncogene Ros1 that displays receptor tyrosin kinase activity. Cancer induction through chromosomal rearrangements that created oncogenic Ros variants has been reported in lung tissue [20].

Finally, network 3 connected Gja3 (gap junction protein, alpha 3) and Gjb3 (gap junction protein, beta 3) to Tp53. The level of gap junction proteins is usually down regulated in cancer cells and it is assumed that the lack of gap junctions is a step in cancerogenesis [21].

1 Bayle JH, Randazzo F, Johnen G, Kaufman S, Nagy A et al. (2002) Hyperphenylalaninemia and impaired glucose tolerance in mice lacking the bifunctional DCoH gene. J Biol Chem 277: 28884-91.

2 Denis M, Landry YD, Zha X (2008) ATP-binding cassette A1-mediated lipidation of apolipoprotein A-I occurs at the plasma membrane and not in the endocytic compartments. J Biol Chem 283:16178-86.

3 Tomimoto S, Ojika T, Shintani N, Hashimoto H, Hamagami K et al. (2008)

Markedly reduced white adipose tissue and increased insulin sensitivity in adcyap1-deficient mice. J Pharmacol Sci 107:41-8.

4 Yamagata T, Yamagata Y, Nakanishi M, Matsunaga K, Minakata Y et al. (2004) A case of primary lung cancer producing alpha-fetoprotein. Can Respir J 11:504-6.

5 Klezovitch O, Chevillet J, Mirosevich J, Roberts RL, Matusik RJ et al. (2004) Hepsin promotes prostate cancer progression and metastasis. Cancer Cell. 6:185-95.

6 Rotondo R, Mastracci L, Piazza T, Barisione G, Fabbi M et al. (2008)

Arginase 2 is expressed by human lung cancer, but it neither induces immune suppression, nor affects disease progression. Int J Cancer 123:1108-16.

7 Motoki K, Megahed M, LaForgia S, Uitto J (1997) Cloning and chromosomal mapping of mouse ladinin, a novel basement membrane zone component. Genomics 39:323-30.

8 Buckbinder L, Velasco-Miguel S, Chen Y, Xu N, Talbott R et al. (1997) The p53 tumor suppressor targets a novel regulator of G protein signaling. PNAS 94: 7868-7872.

9 Zhang H, Kim JK, Edwards CA, Xu Z, Taichman R et al. (2005) Clusterin inhibits apoptosis by interacting with activated Bax. Nature Cell Biol 7: 909-915.

10 Wong GW, Yasuda S, Madhusudhan MS, Li L, Yang Y et al. (2001) Human tryptase epsilon (PRSS22), a new member of the chromosome 16p13.3 family of human serine proteases expressed in airway epithelial cells. J Biol Chem 276:49169-82

11 Mortier E, Wuytens G, Leenaerts I, Hannes F, Heung MY et al. (2005) Nuclear speckles and nucleoli targeting by PIP2-PDZ domain interactions. EMBO J 24:2556-65.

12 Li G, Hu Y, Huo Y, Liu M, Freeman D et al. (2006) PTEN deletion leads to up-regulation of a secreted growth factor pleiotrophin. JBC 16:10663-10668.

13 Wolff JC, Goehring K, Heckmann M, Bux J (2006) Sex-dependent up regulation of CD 177-specific mRNA expression in cord blood due to different stimuli. Transfusion. 46:132-6.

14 Lauritzen I, Chemin J, Honore E, Jodar M, Guy N et al. (2005) Cross-talk between the mechano-gated K2P channel TREK-1 and the actin cytoskeleton. EMBO Rep 6:642-8.

15 Appel S, Filter M, Reis A, Hennies HC, Bergheim A et al. (2002) Physical and transcriptional map of the critical region for keratolytic winter erythema (KWE) on chromosome 8p22-p23 between D8S550 and D8S1759. Europ J Hum Genet 10:17-25.

16 Hart GW, Lowe JB, Sathyamoorthy N (2004) Glycobiology and cancer: meeting summary and future directions. Cancer Biol Ther 3: 233–237.

17 Sakowicz R, Finer JT, Beraud C, Crompton A, Lewis E et al. (2004) Antitumor Activity of a Kinesin Inhibitor. Cancer Res 64:3276-80.

18 Li Y, Ozaki T, Kikuchi H, Yamamoto H, Ohira M et al. (2008) A novel HECT-type E3 ubiquitin protein ligase NEDL1 enhances the p53-mediated apoptotic cell death in its catalytic activity-independent manner. Oncogene 27:3700-9.

19 Wei Y, Liu D, Ge Y, Zhou F, Xu J et al. (2008) Identification of E1AF as a Target Gene of E2F1-induced Apoptosis in Response to DNA Damage. J Biochem 144:539-46.

20 Acquaviva J, Wong R, Charest A (2008) The multifaceted roles of the receptor tyrosine kinase ROS in development and cancer. Biochim Biophys Acta 1795:37-52.

21 Leithe E, Sirnes S, Omori Y, Rivedal E (2006) Downregulation of gap junctions in cancer cells. Crit Rev Oncog 12:225-56.
